# Formulation Strategies to Enhance the Solubility of Poorly Water-Soluble Drugs and Phytochemicals: Current Advances and Challenges

**DOI:** 10.3390/pharmaceutics18050611

**Published:** 2026-05-17

**Authors:** Shery Jacob, Hiral Shah, Anroop B. Nair

**Affiliations:** 1 Department of Pharmaceutical Sciences, College of Pharmacy, Gulf Medical University, Ajman 4184, United Arab Emirates; 2 Department of Pharmaceutics, Parul College of Pharmacy and Research, Parul University, Bopal, Ahmedabad 380058, India; hiral.shah29586@paruluniversity.ac.in; 3 Department of Pharmaceutical Sciences, College of Clinical Pharmacy, King Faisal University, Al-Ahsa 31982, Saudi Arabia; anair@kfu.edu.sa

**Keywords:** poorly aqueous-soluble drugs, phytochemicals, solubility enhancement, micronization, solid dispersion, cocrystallization, cryogenic techniques, solubilization techniques, cyclodextrin complexes, nanotechnology

## Abstract

The low water solubility of numerous drug candidates and phytochemicals continues to pose a significant challenge in pharmaceutical development, greatly limiting their bioavailability and therapeutic performance. This review presents a detailed overview of formulation strategies aimed at improving the solubility and dissolution of poorly aqueous-soluble compounds. The biopharmaceutics classification system and the relevance of in vitro–in vivo correlation, as well as key challenges in formulation development, are briefed. Solid-state and particle engineering approaches, including micronization, supercritical fluid technology, electrospinning, and cryogenic techniques, are discussed. Extensive critical examination of amorphous solid dispersions and their preparation methods, as well as crystallization inhibition strategies, is covered. Cocrystallization is highlighted as a promising approach, with emphasis on design principles and preparation methods. Various solubilization techniques, such as pH modification, cosolvency, hydrotropy, micellar solubilization, and cyclodextrin-based complexation, including advanced hybrid systems, are also explored. Emerging solvent platforms, such as deep eutectic systems and lipid-based and nanotechnology-driven approaches, are reviewed for their role in improving solubility and drug delivery. Additionally, enabling technologies such as liquisolid systems and hydrophilic polymers are addressed. Despite notable progress, limitations such as scalability, reproducibility, regulatory constraints, and long-term safety persist. Overall, this review provides integrated insights into formulation design approaches to enhance the solubility and therapeutic efficacy of poorly soluble drugs.

## 1. Introduction

The biopharmaceutics classification system (BCS) is a scientifically established framework that classifies drug substances into four categories based on their aqueous solubility and intestinal permeability. The primary objective of BCS classification is to predict in vivo oral drug absorption and support regulatory decisions such as biowaivers for immediate-release solid oral dosage forms [1]. According to FDA guidelines, a drug substance is considered highly soluble when the highest dose strength dissolves in 250 mL or less of aqueous buffer media over a physiological pH range of 1–8 at 37 ± 1 °C. A drug substance is regarded as highly permeable if the extent of absorption is ≥85% of the administered dose in humans, as determined from mass balance studies or absolute bioavailability data, in the absence of documented instability in the gastrointestinal tract (GIT) [2]. Based on these criteria, drugs are categorized as class I (high solubility, high permeability), class II (low solubility, high permeability), class III (high solubility, low permeability), or class IV (low solubility, low permeability), with each class having distinct implications for formulation development, absorption rate-limiting steps, and regulatory approval pathways [3]. The BCS is widely adopted by regulatory agencies such as the US FDA, EMA, and WHO, particularly to justify waivers of in vivo bioequivalence studies for class I and, under specific conditions, class III drugs, provided rapid dissolution and appropriate excipient similarity are demonstrated. Despite its broad utility, the BCS does not fully account for factors such as drug metabolism, transporter-mediated effects, or complex dosage forms. Nevertheless, it remains a cornerstone concept in biopharmaceutics, generic drug development, and pharmaceutical regulation.

### Drug Solubility Measurement Techniques and Terminology

Solubility is a critical physicochemical parameter influencing drug absorption and formulation design. Commonly used terms include kinetic, equilibrium (thermodynamic), intrinsic, and apparent solubility. Kinetic solubility refers to the concentration at which a compound begins to precipitate from solution, typically assessed using solvent-shift methods such as dilution from an organic solvent into aqueous media and spectroscopical monitoring [4]. In contrast, equilibrium solubility is determined by adding excess solid drug to a solvent and allowing the system to reach equilibrium, typically over 24–72 h at controlled temperature (e.g., 25 °C or 37 °C), commonly using the shake-flask method followed by analytical quantification [5]. Intrinsic solubility is a specific case of equilibrium solubility and represents the solubility of the unionized (free acid or base) form of an ionizable compound at a pH where it is fully un-ionized, often determined using buffered systems or potentiometric methods [6]. Apparent solubility, on the other hand, describes the total equilibrium solubility at a given pH, including both ionized and unionized forms, and is therefore pH-dependent [7]. Understanding these distinctions is essential for interpreting solubility data. While intrinsic solubility reflects the inherent physicochemical property of a compound, apparent and kinetic solubility are more relevant to physiological conditions and formulation development [8].

## 2. BCS Classification and In Vitro–In Vivo Correlation

The BCS plays an important role in establishing in vitro–in vivo correlation (IVIVC) by linking in vitro dissolution behavior with in vivo drug absorption, thereby helping predict bioavailability and supporting biowaiver decisions during drug development [9]. In case of BCS class II drugs, IVIVC is more likely when dissolution is the rate-limiting step in absorption, typically characterized by a high absorption number and low dissolution number. Furthermore, BCS class II drugs are often subdivided into class IIa (dissolution rate-limited absorption), class IIb (solubility-limited absorption), and class IIc (permeability-limited at high doses due to precipitation or supersaturation effects), which helps better predict the likelihood and quality of IVIVC during formulation development [10]. In BCS class III drugs, the potential for IVIVC is limited because absorption variability is mainly influenced by physiological factors rather than formulation-related effects. IVIVC is typically not achievable for BCS class IV drugs due to their low solubility and permeability, making in vivo bioequivalence studies necessary [11]. However, solubility enhancement approaches for class II and IV drugs, as well as the use of sustained release formulations, can modify absorption characteristics and affect the feasibility of developing IVIVC.

## 3. Challenges in Formulation Development

### 3.1. Challenges Associated with Poorly Water-Soluble Drugs (PWSDs)

The formulation development of PWSDs belonging to BCS class II and IV presents several challenges that can significantly affect therapeutic performance. A major difficulty is the low dissolution rate in gastrointestinal fluids, which often leads to poor absorption and consequently variable oral bioavailability [12]. These drugs may also exhibit problems such as dose limitations, precipitation during formulation or after administration, and instability in supersaturated systems. Therefore, overcoming solubility-related limitations remains a major focus in pharmaceutical formulation research for improving drug dissolution, bioavailability, and therapeutic effectiveness [13].

### 3.2. Solubility Challenges of Phytochemicals

Phytochemicals often present unique challenges in formulation due to their complex chemical structures, poor aqueous solubility, and variable physicochemical properties. Major classes include polyphenols (flavonoids, phenolic acids), terpenoids, alkaloids, glycosides, and lignans. Many widely studied compounds (e.g., curcumin, resveratrol, quercetin, silymarin, naringenin, berberine, genistein, and apigenin) exhibit low solubility and bioavailability due to high lipophilicity, poor dissolution, and susceptibility to degradation [14,15]. Additionally, phytochemicals may exist as complex mixtures or multiple isoforms, and their physicochemical properties can vary depending on plant source, processing, and extraction methods, leading to challenges in solubility characterization and reproducibility. Therefore, solubility enhancement is particularly critical for phytochemicals to ensure consistent performance and optimal therapeutic outcomes [16,17]. Therefore, addressing solubility enhancement is particularly critical for maximizing the therapeutic potential of phytochemicals.

To address these challenges, a variety of formulations and drug delivery strategies have been developed. However, to date, no single review has comprehensively covered all available approaches. Therefore, the present review aims to provide an integrated overview of both conventional and advanced techniques for improving the solubility and permeability of PWSDs. Various formulation techniques employed in enhancing drug/phytochemical solubility are discussed below.

## 4. Solid-State and Particle Engineering Approaches

### 4.1. Particle Size-Reduction Techniques

**Figure 1:**
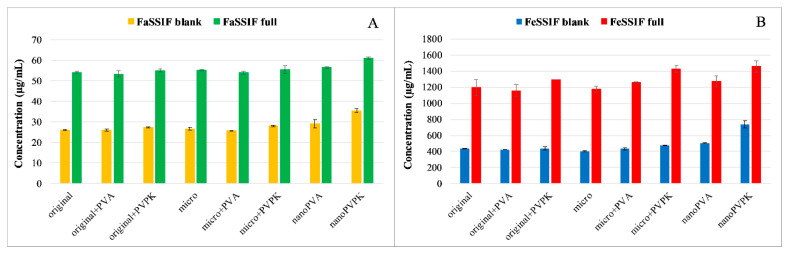
Observed solubility of papaverine hydrochloride in buffers of pH = 6.5 (**A**) and pH 5.0 (**B**). Reproduced from Ref. [22].

Particle size-reduction technologies involve modifying the physicochemical, micrometric, and biopharmaceutical properties of PWSDs to enhance their solubility [18]. Among the numerous techniques for solubility improvement, particle size reduction and crystal habit modification are commonly employed approaches to enhance drug solubility. When the particle size is decreased, the increase in the surface area to volume ratio increases the dissolution rate of the substance as described by the classical Noyes–Whitney equation [19]. According to the Ostwald–Freundlich equation (below), there is a notable increase in solubility when the particle diameter is decreased to less than 1 µm [20].

$$log\;(\frac{C_{S}}{C_{\infty }})=\frac{2\gamma V}{2.303RT\rho r}$$

where $C_{S}$ is saturated solubility, $C_{\infty }$ is the solubility of a solid consisting of large particles, γ is the interfacial tension between the particles and the medium, V is the molar volume of particles, R is the gas constant, T is absolute temperature, ρ is the density of the solid, and r is particle radius. Technologies have been advanced to achieve a reduction in particle size to the nanometer range through the application of nanotechnology and nanosization. These techniques are extensively investigated for their applicability in formulating approaches for PWSDs [21]. Studies using model drugs (e.g., papaverine, furosemide, and niflumic acid) show that micronization enhances dissolution without affecting solubility, whereas nanonization can improve solubility [22]. However, the effectiveness of nanonization depends on the choice of excipients, as certain polymers enhance solubility and prevent particle aggregation more effectively than others, although maintaining particles in the submicron range remains challenging. For instance, nanoformulation with polyvinylpyrrolidone (PVP) K-25 (nanoPVPK) significantly improved the solubility of papaverine hydrochloride, while polyvinyl alcohol (PVA) (nanoPVA) showed no effect (Figure 1). The enhancement is due to the combined effect of particle size reduction and the solubilizing properties of PVPK, rather than nanonization alone.

#### Mechanical Micronization

Mechanical comminution, involving processes such as crushing, grinding, and milling of coarse particles, is widely employed to enhance the solubility of drugs, particularly those classified under BCS class II [23]. Size reduction is typically achieved using frequently employed dry methods such as fluid energy mills (jet mills) and ball mills, as well as widely used wet milling techniques such as media milling (nanomilling). The size-reduction mechanisms occur through pressure, friction, compression, attrition, impact, or shearing. Traditional particle size-reduction techniques, particularly milling, have several limitations, including high energy requirements, risk of thermal or chemical degradation of drugs, and production of particles with broad and non-uniform size distribution. In addition, these methods are often considered relatively uncontrolled, making it difficult to precisely regulate particle characteristics such as size, shape, surface morphology, and electrostatic properties [24]. Recent advances, such as mini-scale milling devices with temperature control, enable efficient nanocrystal production from small drug quantities (e.g., itraconazole, ivermectin, and curcumin) while maintaining stability and scalability. These systems also show no adverse effects on milling materials or induce polymorphic changes in the nanocrystals, as confirmed by calorimetric analysis [25].

### 4.2. Advanced Particle Engineering

Particle engineering techniques, including cryogenic spray and crystal engineering processes, have been devised as innovative methods to produce nanosized drug particles [26]. Among these methods, cryogenic processes such as cryo-milling, spray freeze drying (SFD), spray freezing into cryogenic liquid (SFCL), and thin-film freezing (TFF) are widely used to produce nanosized drug particles. These techniques involve rapid freezing of the drug solution in a cryogenic medium, which induces rapid nucleation and prevents crystal growth. As a result, amorphous or nanostructured particles with very high surface area are formed, leading to enhanced dissolution rates and supersaturation in biological fluids. The increased surface area and porous structure of the particles significantly improve drug dissolution and may enhance the oral bioavailability of PWSDs. In addition, stabilizing polymers are often incorporated during the process to prevent particle aggregation and maintain the nanosized structure of the drug particles [27]. Another important particle engineering strategy is crystal engineering, which involves modifying the crystal structure and intermolecular interactions of active pharmaceutical ingredients (APIs) to improve their physicochemical properties [28]. Approaches such as cocrystal formation, polymorph modification, salt formation, and crystal habit modification can enhance the solubility and dissolution rate of drugs. Crystal engineering enables rational design of drug crystals by controlling molecular packing and lattice energy, thereby enhancing dissolution behavior without altering the pharmacological activity of the drug. Studies have demonstrated that techniques such as nano-cocrystals and multicomponent crystal systems can substantially increase kinetic solubility and dissolution rates, ultimately improving bioavailability of PWSDs [29].

#### 4.2.1. Supercritical Fluid (SCF) Technology

The SCF technique, most commonly using supercritical carbon dioxide (scCO_2_), is an advanced method for preparing solid dispersions (SDs), particularly amorphous SDs (ASDs) [30]. SCF is a state maintained above its critical temperature and pressure, where it exhibits both gas-like diffusivity and liquid-like solvating power. In pharmaceutical applications, scCO_2_ is favored because it is non-toxic, non-flammable, inexpensive, and easily removed after processing. In the supercritical anti-solvent method, the drug and polymer are dissolved in an organic solvent, and scCO_2_ acts as an anti-solvent. The scCO_2_ rapidly diffuses into the solution and reduces solvent power, leading to coprecipitation of the drug and carrier as fine particles, often in an amorphous state [31]. In the rapid expansion of supercritical solutions, the drug is first dissolved in scCO_2_ and then rapidly depressurized through a nozzle, causing supersaturation and particle formation, although its application is limited by the low solubility of many drugs and polymers in scCO_2_ [32]. Another approach is supercritical fluid impregnation, where scCO_2_ plasticizes and swells the polymer matrix, allowing the drug to diffuse into it; upon depressurization, SD may be formed [33].

SDs prepared using SCF techniques typically exhibit small particle size, narrow particle size distribution, high surface area, reduced crystallinity or complete amorphization, and low residual solvent content [34]. Key advantages of the SCF technique include minimal thermal degradation, reduced use of harmful organic solvents, improved control over particle morphology and size through adjustment of pressure and temperature, and the possibility of combining particle formation and drying in a single step [35]. However, the solubility of many drugs and polymers in scCO_2_ is low, restricting their applicability. Supercritical antisolvent-based methods still require organic solvents, and careful solvent selection is essential [36]. High-pressure equipment increases operational complexity and cost, and scale-up can be challenging. Additionally, ASDs produced by SCF methods may face physical stability issues, such as recrystallization during storage [37]. Ongoing research focuses on process optimization, continuous manufacturing, improved nozzle design, and a better understanding of phase behavior to enhance the scalability and long-term stability of SCF-produced SDs [30].

ASD nanoparticles (750 nm) of the poorly soluble antibiotic suladiazine were successfully prepared using PVP as a polymeric carrier through the supercritical antisolvent process [38]. A systematic three-stage investigation covering pure sulfadiazine, pure PVP, and the combined sulfadiazine/PVP system was conducted to determine the optimal operating conditions and evaluate the influence of key process parameters on nanoparticle formation. The produced formulation demonstrated a significantly enhanced dissolution rate compared to the corresponding physical mixture. The SCF technique was evaluated as a green alternative for preparing nifedipine SDs and compared with the kneading method [39]. The SCF formulation with a higher polymer ratio (1:5) produced fully amorphous nifedipine, showed the fastest dissolution, and exhibited altered pharmacokinetic behavior compared to the conventional formulation. Another comparative study conducted by the same author evaluated the improvement in solubility, dissolution rate, and oral bioavailability of nifedipine using polyethylene glycol (PEG) 4000-based SDs (1:1, 1:3, and 1:5 ratios) prepared by SCF technique and kneading methods [40]. SCF technique produced amorphous dispersions with superior performance, particularly the 1:3 formulation, which demonstrated a 3.34-fold increase in bioavailability and an increase in Cmax from 0.78 ± 0.27 to 2.26 ± 0.32 μg/mL.

SCF minimizes solvent residue issues by replacing organic solvents with high-pressure CO_2_, and the solubility of the drug in CO_2_ is the key factor influencing formulation success. SCF technique using CO_2_ was employed to prepare albendazole SDs to improve its solubility and dissolution using Kollidon^®^ VA 64 [41]. Solid-state characterization confirmed significant amorphization of the drug in SCF-processed batches prepared at 80 °C and 1500 psi for 2 h. The SCF formulation demonstrated improved dissolution and a two-fold increase in permeability compared to the pure drug, confirming its effectiveness in improving the biopharmaceutical performance of albendazole.

SCF technology offers significant advantages in particle engineering and solid-state modification of APIs. By carefully controlling process parameters such as pressure, temperature, and flow rate, SCF techniques enable precise tailoring of particle morphology and reproducible control over the polymorphic form of crystalline APIs [42]. These processes typically yield free-flowing, micronized powders with reduced interparticle adhesion and cohesion, improving powder dispersibility and making them particularly suitable for aerosolization in pulmonary drug delivery systems [43]. Furthermore, SCF-based micronization operates under relatively mild thermal conditions, making it especially advantageous for thermally sensitive materials, including recombinant proteins and monoclonal antibodies [44]. From a commercial perspective, SCF micronization is generally more appropriate for high-value drug delivery applications like inhalation therapies or specialized formulations rather than conventional oral solid dosage forms [45].

#### 4.2.2. Electrospinning Technique

Electrospinning represents a promising and adaptable approach for enhancing the solubility and permeability of BCS class II and IV drugs. The formation of nanofibers with high surface area and amorphous drug dispersion significantly improves dissolution rates and apparent solubility [46]. Although challenges related to scale-up and stability remain, electrospun nanofibers offer strong potential as an advanced platform for overcoming biopharmaceutical limitations of poorly soluble and permeable drugs. Electrospinning is a fiber-forming technique in which a polymer solution is exposed to a high-voltage electrostatic field, causing the solution to stretch and form a fine jet from the tip of the Taylor cone [47]. As the jet travels toward the collector, rapid solvent evaporation occurs, resulting in the formation of solid polymeric fibers. This fast solidification limits drug molecular mobility, inhibits crystallization, and promotes random molecular dispersion of the drug within the polymer matrix. The resulting fibrous mats exhibit a high surface-to-volume ratio, which enhances dissolution performance. Additionally, the intimate drug–polymer mixing and rapid solvent removal facilitate the formation of physically stable ASDs. These fibrous mats can be milled or directly converted into oral dosage forms, and they are characterized by very high surface area, porous nonwoven structure, short diffusion pathways for water ingress, and strong drug–polymer interactions that help sustain supersaturation during dissolution [48].

Optimization of electrospinning conditions has been extensively investigated to produce nanofibers with controlled morphology and reproducible properties. The influencing factors are generally categorized into three groups, namely solution parameters (e.g., polymer molecular weight and concentration, conductivity, surface tension, and solvent type), process parameters (e.g., flow rate, applied voltage, needle-collector distance, needle diameter, and collector type), and ambient conditions (e.g., temperature, relative humidity, and air flow) [49]. Commonly used polymers in pharmaceutical electrospinning include biodegradable and biocompatible materials such as poly(lactic acid), polycaprolactone, poly(lactic-co-glycolic acid), PVA, PVP, PVA/VA copolymer, polyethylene oxide, cellulose derivatives (e.g., cellulose acetate), and natural polymers like chitosan, alginate, collagen, and gelatin, owing to their suitable molecular weight, spinnability, and compatibility for drug delivery and tissue engineering applications [50].

**Figure 2:**
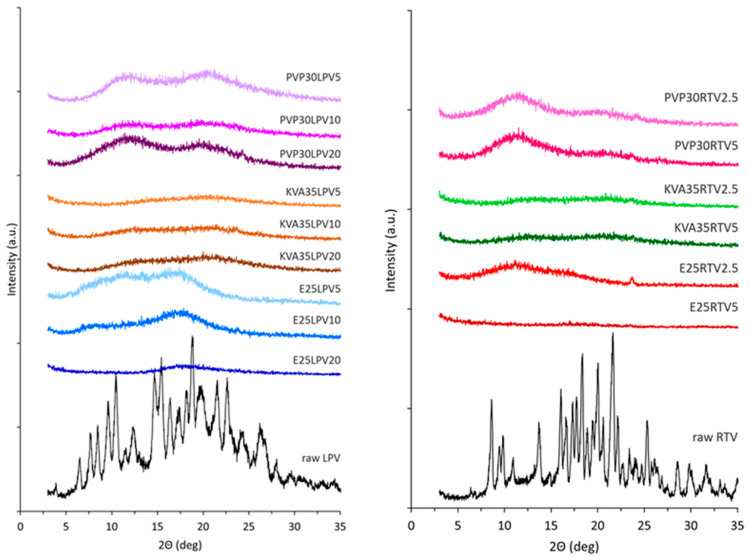
Representative X-ray diffraction patterns of raw lopinavir (LPV)/lopinavir-loaded electrospun fibers (**left**) and raw ritonavir (RTV)/ritonavir-loaded electrospun fibers (**right**). Reproduced from Ref. [53].

Electrospun ASDs of flubendazole with water-soluble synthetic polymer, poly(2-ethyl-2-oxazoline), were developed to enhance bioavailability, achieving ultra-high drug loading (up to 55 wt%) and stability for at least one year [51]. Strong drug–polymer hydrogen bonding enabled high incorporation and maintained the amorphous state. At high drug loadings, the polymer concentration had to be reduced below its usual spinnable limit due to increased viscosity from drug–polymer interactions. The formulations showed marked performance improvement, with solubility and dissolution increasing three- and four-fold, respectively, compared to the crystalline drug. The feasibility of producing ultra-high drug-loaded ASDs of class II drugs, including itraconazole, mebendazole, celecoxib, and fenofibrate, using solvent electrospinning with poly(2-ethyl-2-oxazoline) or PVP was demonstrated [52]. By reducing polymer concentration below its typical spinnability threshold, ASDs containing up to 80 wt% drug were successfully prepared. High drug loading was attributed to favorable drug–polymer interactions beyond hydrogen bonding, including Van der Waals forces. Electrospinning was used to prepare ASDs of the poorly soluble antiretroviral drugs lopinavir and ritonavir [53]. Using different polymer carriers (PVP, Kollidon-Vinyl Acetate, and Eudragit^®^ E100), complete drug amorphization was achieved regardless of drug loading. Eudragit improved lopinavir performance, while Kollidon copolymer enhanced ritonavir solubility and dissolution. X-ray diffraction (XRD) analysis (Figure 2) confirmed the transformation of crystalline drugs into an amorphous state after electrospinning. While raw lopinavir and ritonavir showed sharp Bragg peaks indicative of crystallinity, the electrospun formulations exhibited diffuse amorphous halos, demonstrating complete amorphization irrespective of drug content or polymer type.

The poor bioavailability of hesperidin, resulting from its low solubility and permeability, was addressed by preparing electrospun nanofibers composed of PVP and hydroxypropyl-β-cyclodextrin (HP-β-CD) [54]. These nanofibers, loaded with hesperidin-rich extract, showed more than eight-fold increase in solubility and over nine-fold improvement in transmucosal permeation. The formulated nanofibers also demonstrated strong antioxidant and anti-inflammatory activities, particularly those based on HP-β-CD and PVP.

#### 4.2.3. Cryogenic Techniques

Among cryogenic techniques, cryogenic grinding (cryo-milling), spray-freezing into cryogenic liquid (SFCL), SFD, and TFF are increasingly used to increase the solubility and bioavailability of BCS class II and IV drugs [27]. These bottom-up precipitation processes induce amorphous, ultrafine, or nanostructured particles, thereby increasing specific surface area, enhancing wettability, promoting higher dissolution rates, and inducing supersaturation. This is achieved by rapid nucleation followed by polymer-stabilized growth arrest and solvent solidification, often yielding partially or fully amorphous high-energy solids with enhanced dissolution and apparent solubility. For class II drugs, faster dissolution frequently translates directly into higher oral exposure, while for class IV drugs, cryogenic processing is often paired with enabling excipients (e.g., surfactants, polymers, lipid carriers) to address both dissolution and permeation constraints.

##### Cryo-Milling

**Figure 3:**
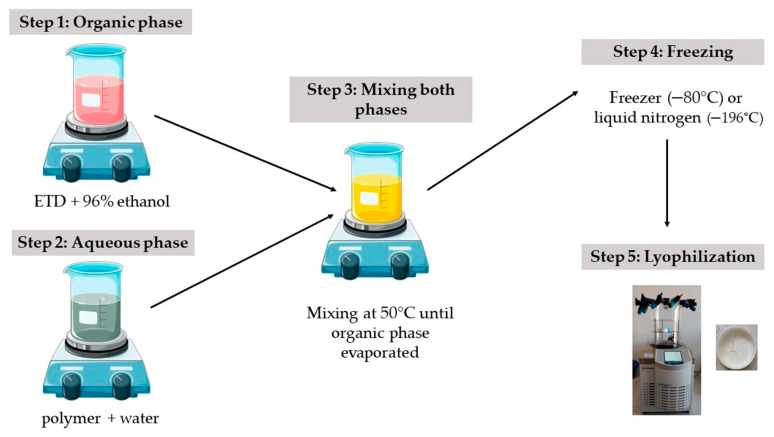
Preparation method of etodolac by the lyophilization technique. Reproduced from Ref. [60].

Drug amorphization during cryo-milling occurs due to intense mechanical stress, which induces particle size reduction, polymorphic transitions, structural disorder, and partial or complete conversion to the amorphous state [55]. For instance, ultra-cryogenic milling using liquid nitrogen produced finer and more uniform phenytoin particles than jet milling, but did not improve dissolution due to particle agglomeration [56]. However, co-grinding phenytoin with PVP significantly enhanced dissolution by improving wettability and dispersion without altering the crystalline structure of the drug. Similar improvement was observed when PVP was ground separately and then mixed, indicating independent grinding behavior. Co-grinding with other excipients also enhanced dissolution, demonstrating the broad applicability of the ultra-cryo milling technique for PWSDs. Spherical D-mannitol beads were developed as a safe alternative to conventional milling beads, which suffer from wear and lack ingestible safety [57]. Co-milling with this spherical sugar reduced phenytoin particle size significantly with increasing bead amount and agitation speed, while milling time had minimal effect. In another study, spherical crystalline cellulose, a common pharmaceutical excipient, was evaluated as a safe alternative to conventional milling beads [58]. Milling of phenytoin in liquid nitrogen using spherical cellulose produced particle sizes comparable to zirconia beads, with D50 values as low as 0.3 μm. Catalytic pretreated softwood cellulose and PVP were evaluated as carrier polymers for preparing cryogenic co-ground ASDs of piroxicam [59]. The choice between cellulose and PVP significantly influenced drug amorphization, stability, and release behavior. PVP enhanced the dissolution rate, whereas softwood cellulose provided a sustained release effect, highlighting the critical role of polymer selection in determining the performance of cryogenically prepared SDs. APIs and polymers may undergo degradation during milling, especially in combination, indicating that cryo-milling is not a mild technique for ASD preparation. SDs of etodolac were developed using hydrophilic and amphiphilic polymers to improve its solubility and dissolution [60]. Poly(vinylpyrrolidone-vinyl acetate) (PVP/VA), HPMC, and poloxamer were used as carriers, and SDs were prepared by cryo-milling and lyophilization. To prepare SDs using the lyophilization technique, etodolac was dissolved in ethanol and the polymer in water, followed by mixing under stirring and heating to achieve partial solvent evaporation. The resulting dispersion was then frozen either rapidly using liquid nitrogen or at −80 °C and lyophilized under controlled conditions with an additional drying step (Figure 3). Lyophilized formulations containing PVP/VA and/or poloxamer showed the best performance by enhancing dissolution, reducing crystallinity, and preventing recrystallization through hydrogen bonding interactions.

##### Spray-Freezing into Cryogenic Liquid (SFCL)

SFCL is a simple and efficient cryogenic technique in which drug formulations are atomized into compressed or cryogenic liquids, leading to rapid freezing and formation of nanostructured particles that are converted into free-flowing nanopowders after lyophilization. This method has been shown to significantly enhance drug dissolution, as demonstrated with danazol compared to conventional size-reduction approaches [61]. Glimepiride, a BCS class II drug, was formulated as nanostructured particles using the SFCL technique with PVP K-30, resulting in significantly improved solubility and in vitro drug release [62]. The optimized formulation exhibited high production yield, nanoparticles (280 ± 62 nm to 520 ± 30 nm), significant hypoglycemic activity in diabetic rats, and a 1.79-fold increase in bioavailability compared with marketed tablets. Key advantages include processing at very low temperatures that can reduce thermal degradation, enable formation of porous, highly dispersible powders, and facilitate co-amorphous/co-processed systems (drug–polymer or drug–coformer) with improved dissolution. However, limitations include higher operational costs and process complexity due to the need for cryogens and specialized safety infrastructure, as well as challenges in scale-up and yield [63]. Critically, there is also a risk of physical instability, such as amorphous recrystallization, moisture sensitivity, and particle agglomeration that can reduce performance over shelf life. In addition, class IV drugs may demonstrate limited bioavailability improvement unless permeability is concurrently enhanced.

##### Thin Film Freezing (TFF)

TFF is a rapid freezing technique used to produce brittle, highly porous, low-density drug particles [64]. In this method, the drug solution is spread as a thin film over a cryogenic surface, leading to ultra-rapid solidification that minimizes crystal growth and often results in amorphous or nanostructured materials. The resulting frozen matrix is subsequently lyophilized to obtain dry powders with enhanced surface area and improved dissolution properties. TFF is particularly advantageous for PWSDs and for developing inhalable dry powder formulations due to its ability to generate particles with excellent aerosolization performance. Recently, machine learning models were successfully developed to predict aerosol performance (fine particle fraction) and mass median aerodynamic diameter of dry powder inhalers produced by TFF [65].

##### Spray-Freeze Drying (SFD)

SFD has recently emerged as a promising alternative to conventional freeze drying and spray drying, as it produces powders with unique properties that enhance drug performance. In particular, SFD is effective in producing biopharmaceuticals with improved stability and solubility [66]. SFD was employed to enhance the solubility of poorly water-soluble cannabidiol for pulmonary delivery [67]. Formulations containing mannitol or trehalose, with dipalmitoylphosphatidylcholine, produced porous particles with significantly improved solubility, increasing from 0.36 µg/mL to over 2 µg/mL. The improvement was attributed to reduced particle size and increased porosity induced by phospholipid. Current innovations focus on integrating cryogenic techniques with ASDs, co-amorphous systems, and hybrid platforms, alongside better stabilization strategies using polymers and optimized residual moisture control [68]. A representative pharmaceutical example is celecoxib (class II) formulated via SFD with hydrophilic carriers (e.g., PVP), producing porous amorphous or partially amorphous powder with markedly faster dissolution and improved exposure compared with the crystalline drug [69].

## 5. Amorphous Solid Dispersion Systems

### 5.1. Solid Dispersion (SD) Systems

It is considered one of the most promising and practical strategies for enhancing the solubility of PWSDs. SD is defined as a system in which one or more hydrophobic APIs are dispersed within an inert carrier or a hydrophilic matrix [70]. Primary carriers in SDs include sugars and polyols (e.g., mannitol), water-soluble polymers (e.g., PVP), enteric/pH-dependent polymers (e.g., Eudragit^®^ L100), surfactants (e.g., Poloxamer 188), lipid-based carriers (e.g., Gelucire^®^), and low molecular weight carriers (e.g., urea), while co-formers such as organic acids (e.g., citric acid) enhance solubility (Table 1). SDs can be classified into several types based on the physical state and molecular arrangement of the drug within the carrier. These include simple eutectic mixtures, solid solutions, glass solutions, glass suspensions, amorphous precipitates in crystalline carriers, molecular complexes (compounds), and other more complex systems [71]. SD adsorbate was also used to enhance the solubility of PWSDs [72].

Solubilization is primarily achieved by eliminating or markedly reducing drug crystallinity and promoting molecular-level dispersion of PWSDs within a hydrophilic polymeric matrix. Upon exposure to aqueous media, the hydrophilic carrier dissolves and releases the drug as fine colloidal particles. Reduced particle size increases surface area, enhancing dissolution rate, while improved wettability and matrix porosity promote solvent penetration. Partial amorphization may further increase apparent solubility, thereby maintaining enhanced dissolution performance [95]. However, such high-energy, supersaturated states are thermodynamically unstable and susceptible to drug recrystallization during dissolution and storage. To counteract this, surfactants are incorporated to lower interfacial tension, enhance wetting, and stabilize dispersed particles through steric or electrostatic mechanisms, thus inhibiting nucleation and crystal growth. Surfactants also facilitate micellar solubilization of PWSDs, which further increases apparent solubility, thereby improving overall dissolution performance [96]. Studies report that surfactant inclusion in ASDs or colloidal systems significantly enhances drug release, though careful selection is required to balance solubilization benefits with physical stability considerations [97]. For instance, the incorporation of surfactants significantly improved drug release from ritonavir-PVP/VA ASDs compared with the binary system. This improvement is attributed to enhanced drug–polymer water miscibility and surfactant-induced plasticization, which disrupts the drug-rich interfacial barrier at the gel–solvent interface.

#### 5.1.1. Eutectic Systems

Eutectic systems in SDs are mixtures of PWSD and carrier that are fully miscible in the liquid state but exhibit limited solid-state miscibility. Upon cooling, they solidify into two finely dispersed crystalline phases or a solid solution at a specific eutectic composition and temperature [98]. Eutectic systems display a melting point lower than that of their individual components, resulting from intermolecular interactions, lattice disruption, and increased entropy. Modern formulation strategies utilize crystal engineering concepts, including supramolecular synthons, to optimize eutectic systems and improve solubility, stability, and drug efficacy. Eutectic formulations can improve the solubility of BCS class II and IV drugs and may also enhance the permeability of class III and IV drugs. These effects are primarily attributed to non-covalent interactions, such as hydrogen bonding and π-π stacking, between the drug and the carrier. For example, hydrogen bonding in the ibuprofen–menthol eutectic system enhances solubility and permeability, thereby enhancing bioavailability and potentially shifting the drug from BCS class II to class I [99]. A study investigated three ibuprofen–menthol therapeutic eutectic systems prepared at different molar ratios to enhance drug formulation and delivery [100]. The systems formed homogeneous, monophasic, viscous liquids with temperature-dependent physicochemical properties, while viscosity exhibited non-Newtonian flow behavior relevant to pharmaceutical processing.

To formulate a stable and optimized eutectic system, it is essential to understand how the physicochemical and biopharmaceutical characteristics of solid mixtures differ from those of the individual drug components. A 1:1 glimepiride–arginine eutectic mixture (eutectic temperature 426.9 K) improved the drug dissolution by enhancing its solubility and wettability [101]. Gliclazide–succinic acid eutectic system prepared using liquid-assisted grinding and electrospray deposition showed improved dissolution at pH 1.2 relative to pure drug [102].

In recent years, there has been a notable increase in research exploring eutectic mixtures as a strategy to modify physicochemical and biopharmaceutical properties of drugs. Many investigations highlighted the potential of eutectic mixtures produced via hot-melt extrusion (HME) [103]. These systems have been formulated into oral, transdermal, implantable, and mucosal dosage forms, offering controlled release, enhanced bioavailability, and good biocompatibility. A resveratrol–nicotinamide binary eutectic system enabled HME at a lower temperature (155 °C instead of 215 °C), reducing thermal degradation of resveratrol while maintaining dissolution performance [104]. This study highlights the potential of binary eutectic systems in improving the thermal stability of heat-sensitive drugs during ASD processing. A binary eutectic SD of nevirapine and paracetamol (1:3 ratio) was prepared using the solid-state grinding method [105]. The resulting system exhibited 3.07-fold enhancement in dissolution rate compared to pure nevirapine and other multicomponent forms, including the nevirapine–trimeric acid cocrystal. Various organic additives, including xanthine derivatives, hydroxybenzoic acid and its derivatives, as well as vitamins, have been employed as hydrophilic carriers in the development of binary pharmaceutical systems. Among BCS class II drugs, several binary eutectics such as diacerein–2,4-dihydroxybenzoic acid (2.5-fold) [106], nimesulide–nicotinamide (4.7-fold) [107], lovastatin–benzoic acid (~5-fold) [108] and rivaroxaban–caffeic acid (1.5-fold) [109] demonstrated a notable enhancement in dissolution rate. Additionally, the class IV hydrochlorothiazide–atenolol eutectic exhibited a 10-fold improvement in dissolution performance [110]. Mechanochemical screening of trimethoprim eutectic systems, especially with paracetamol combination with GRAS-listed coformers, showed enhanced dissolution performance [111].

#### 5.1.2. Glassy Solution

They are homogeneous amorphous systems with the drug dissolved in a glassy carrier, while glass suspensions contain dispersed precipitated particles [112]. They have low lattice energy, no sharp melting point, and commonly use carriers such as urea, PEG, PVP, citric acid, and sugars. In SDs, the preferred physical state is the glassy (amorphous) state, as it enhances stability and performance. Temperature changes influence the heat content and molar volume of the drug, showing a gradual variation until the glass transition temperature (Tg) is reached, where a sudden shift occurs. Below Tg, the material exists in a rigid glassy state, while above Tg, it transitions into a more flexible rubbery state [113]. The resistance of glasses to devitrification upon reheating reflects their stability in the amorphous state, particularly near or slightly above the Tg [114].

### 5.2. Method of Preparation

Multiple techniques such as fusion, HME, coprecipitation, solvent evaporation, spray drying, and kneading are available for preparing SDs. However, the most suitable method depends on drug-specific properties such as hydrophilicity, dose, and molecular weight. Determining the optimal drug-to-coformer ratio often requires a trial-and-error approach, as it involves systematically evaluating different proportions to achieve desirable physicochemical properties, improved stability, and enhanced dissolution performance.

#### 5.2.1. Fusion Method

In this technique, a physical mixture of the drug and polymer is heated until it forms a homogeneous molten mass. The melt is then cooled to obtain a solidified product, which is subsequently size-reduced by pulverization, grinding, or milling to achieve the desired particle size [115]. Adequate polymer–drug miscibility at elevated temperatures without thermal degradation is essential. Additionally, the formulation must resist recrystallization and phase separation to ensure long-term stability. Several studies have demonstrated significant improvements in solubility and drug release using the fusion method. Luteolin, a poorly water-soluble bioactive natural flavonoid with low oral bioavailability, was formulated as SDs using PEG 4000 at drug-to-polymer ratios of 1:1, 1:2, and 1:4 [116]. A similar effect was reported for valdecoxib as well [117]. Dissolution studies demonstrated significantly enhanced drug release from all solid dispersions compared to the pure drug and its physical mixture. This improvement was primarily attributed to increased aqueous solubility, reduced particle size, and transformation of the drug from a crystalline to an amorphous state. An effervescent-assisted fusion method using Soluplus^®^ and effervescent base demonstrated significantly higher solubility and dissolution of aceclofenac compared to the conventional fusion method [118]. Although both methods converted the drug into an amorphous form, the effervescent method was more effective in enhancing drug release. SDs of haloperidol were prepared using solvent evaporation and melting methods, with PEG 4000 selected as the most suitable polymer [119]. Fast-dissolving tablets prepared from the optimized formulation (SD2), developed by the melting method, showed superior performance, including rapid disintegration, acceptable hardness and friability, enhanced dissolution, and thermodynamic stability. In vivo studies demonstrated greater antipsychotic activity compared to the commercial product.

#### 5.2.2. Hot-Melt Extrusion (HME)

Over the past decade, HME has become an effective technique for enhancing drug bioavailability and developing sustained or modified drug delivery systems [120]. In this method, the drug and polymer are blended and subjected to elevated temperature and pressure inside an extruder, where they are melted, mixed uniformly, and extruded to form SDs with improved homogeneity and dissolution characteristics. This method enables the molten drug–polymer blend to be shaped into various dosage forms and requires complete drug–polymer miscibility predicted using solubility parameters [121]. It offers advantages such as fewer processing steps, continuous and scalable manufacturing, and uniform molecular-level dispersion of the drug [122]. Commonly used polymers in HME include HPMC, hydroxypropyl methylcellulose acetate succinate (HPMCAS), HPC, ethyl cellulose, PVP, copovidone, PVA, PEG, polyethylene oxide, Eudragit^®^ grades, poloxamers, and Soluplus^®^ [123]. SDs of carbamazepine were prepared using HPMC HME 15LV polymer via HME with either single carriers (PVP/VA 64 or Soluplus) or a binary carrier system comprising HPMC HME 15LV combined with Soluplus [124]. The physical state characterization of the drug showed that the drug existed in amorphous form in all formulations. Compared to single-carrier systems, the binary carrier system demonstrated reduced moisture absorption and improved thermal stability, thereby enhancing long-term stability. HME was used to develop ASDs of nintedanib with Soluplus^®^ and HPMCAS to overcome its poor intestinal dissolution and low oral bioavailability [125]. The ASDs prepared by HME exhibited significantly enhanced dissolution (10–16-fold) and improved oral bioavailability (2.5–7.9-fold) compared to the crystalline drug, leading to superior antifibrotic efficacy without significant toxicity. One study assessed a strategy to manufacture high drug-loaded ASDs using an HME platform using three model drug–polymer systems (indomethacin–Eudragit^®^ E, naproxen–Eudragit^®^ E, and ibuprofen–Eudragit^®^ E) [126]. The formulation design space was predicted using Flory–Huggins’ theory, and selected formulations were prepared by HME and quench-cooled melt methods. The results confirmed successful production of ASDs with high drug loadings of 65% (indomethacin), 70% (ibuprofen), and 60% (naproxen). Furthermore, the formulations demonstrated improved physical stability under high humidity conditions (95% RH). In a comparative study, ASDs with HPMCAS were prepared using both HME and spray drying [127]. The addition of TPGS improved extrudability while maintaining comparable solid-state properties. Although spray-dried ASDs demonstrated higher drug release due to smaller particle size, optimizing the particle size of melt-extruded ASDs resulted in similar in vitro and in vivo performance, supporting the feasibility of HME scale-up during drug product development. KinetiSol^®^ technology is a high-energy, solvent-free thermokinetic process used to manufacture ASDs without external heat, making it suitable for thermally sensitive drugs and compounds with challenging physicochemical properties [128].

#### 5.2.3. Solvent Evaporation

In this method, both the drug and a suitable hydrophilic carrier, such as PVP, PEG, or HPMC, are dissolved in a common volatile solvent (e.g., ethanol, methanol, acetone) to form a homogeneous solution. The solvent is then removed by evaporation, vacuum drying, spray drying, or freeze drying, leaving behind SD in which the drug is molecularly dispersed within the carrier matrix. This method is particularly suitable for thermostable drugs and helps improve the dissolution rate of PWSDs. However, complete removal of the solvent is essential to avoid residual solvent issues, and scale-up may be challenging. The solvent-evaporation method for SD offers the advantage of avoiding thermal degradation of drugs and polymers. However, the difficulty in dissolving both drug and polymer in a common solvent, especially when their polarities differ, and the risk of phase separation during solvent removal, remains a challenge [129]. A study on furosemide showed that solvent evaporation provided better solubility enhancement compared to kneading, physical mixtures, and coprecipitation. Dutasteride SDs were prepared by the solvent evaporation method using solubilizer (tocofersolan/TPGS), solvent (methylene chloride), and carriers (Aerosil^®^ 200 or microcrystalline cellulose), and evaluated [130]. The optimized formulations (F15 and F16) showed significantly higher dissolution after 1 h (86–95%) compared to the commercial product Avodart^®^ (≈76%). They also demonstrated improved relative bioavailability (126–132%), likely due to hydrogen bonding between drug and solubilizer. ASDs of lumefantrine were prepared by solvent evaporation using a blend of microcrystalline cellulose and anhydrous lactose, with various polymers mostly containing acidic groups and different drug loadings [131]. Results indicated that an optimal balance between physical stability and dissolution enhancement is necessary in selecting suitable polymers for ASDs. Luteolin was formulated as SD using PVP by the solvent-evaporation method to enhance its bioavailability [132]. PVP K-40 was identified as the optimal carrier, increasing solubility by about 250-fold without affecting stability or activity. The formulation reduced drug crystallinity and demonstrated improved anti-inflammatory effects and enhanced glucose tolerance and insulin sensitivity in mice. The improvement in solubility and bioavailability of dolutegravir by SD using Poloxamer 407, when compared to the pure drug, was demonstrated [133]. Another study assessed the solubility and dissolution rate of bilastine by preparing SDs using various hydrophilic carriers [134]. The best results were obtained using PVP K-30 at a 1:15 ratio via solvent evaporation, achieving a 10.2-fold increase in solubility.

#### 5.2.4. Coprecipitation

In the coprecipitation method, both the drug and the polymer are first dissolved in a common solvent to form a uniform solution. An antisolvent is then introduced to induce precipitation of the ASD [135]. From a scientific perspective, coprecipitation promotes molecular-level mixing between drug and carrier, often leading to reduced crystallinity or formation of an amorphous state. This improves wettability, reduces particle size, enhances drug dispersion, and promotes intermolecular interactions, collectively improving dissolution. This strategy enables both drug amorphization and nanosizing in a single process. When only amorphous polymers are used to molecularly disperse the drug via coprecipitation, second-generation ASDs are formed [136]. The inclusion of surfactant to enhance dissolution and minimize precipitation or recrystallization leads to the development of third-generation ASDs. A comparative study was conducted to prepare ASDs of Compound A in HPMC using HME and solvent coprecipitation [137]. The ASDs prepared via coprecipitation exhibited higher porosity and larger surface area, resulting in faster dissolution, whereas the HME product demonstrated a higher intrinsic dissolution rate and greater stability in aqueous suspension.

**Figure 4:**
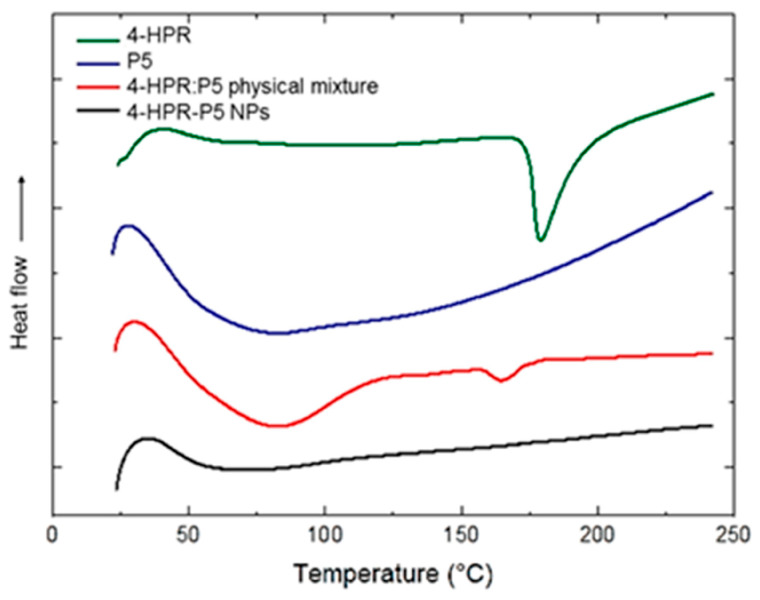
Representative differential scanning calorimetry profiles of raw fenretinide (4-HPR), solid dispersion (P5), the 4-HPR-P5 physical mixture, and 4-HPR-P5 nanoparticles (NPs). Reproduced from Ref. [138].

Fenretinide (4-HPR) has strong antitumor activity but poor clinical performance due to low solubility and high first-pass metabolism [138]. SD prepared by antisolvent coprecipitation with hydrophilic copolymer (P5) increased its apparent solubility by 1134-fold, enhanced dissolution, and formed stable nanoparticles with high drug loading. Differential scanning calorimetry thermograms of raw 4-HPR, P5, the 4-HPR-P5 physical mixture, and 4-HPR-P5 nanoparticles were analyzed to assess the physical state of the drug (Figure 4). The disappearance of the characteristic melting peak of 4-HPR in the nanoparticles formulation, compared to its presence in the pure drug and physical mixture, confirms successful amorphization. Additionally, the absence of a dehydration peak of the polymer suggests improved molecular dispersion of the drug within the polymer matrix, further supporting the formation of an amorphous system. The formulation maintained significant antiproliferative activity, indicating improved bioavailability and therapeutic potential.

A study investigated the effect of different preparation methods on the mechanical properties of ASD of the developmental compound (GDC-0810) formulated with HPMCAS [139]. The results showed that coprecipitated ASD powders exhibited superior flowability and tabletability compared to spray-dried powders. Additionally, resonant acoustic mixing was identified as a scalable and promising approach for producing ASDs through coprecipitation. The coprecipitation method was employed to prepare ASD of vemurafenib with HPMCAS, which was subsequently commercialized under the trade name Zelboraf^®^ [140]. Co-processing of APIs, including direct coprecipitation of ASDs during the final stage of drug substance manufacture, is emerging as a strategy to simplify manufacturing and enhance product stability and bioavailability [141]. ASDs incorporating either high-Tg drug (hydrochlorothiazide) or low-Tg drug (simvastatin) within PVP/VA 64 or Soluplus^®^ polymer matrices were produced using both coprecipitation and spray-drying techniques. Both methods produced amorphous and physically stable ASDs. However, differences were observed in Tg, particle size, and morphology, which may impact downstream processing. Additionally, coprecipitation of simvastatin required high antisolvent ratios, highlighting practical considerations when selecting between processes. Microprecipitated bulk powder is another SD technology designed to produce amorphous formulations of PWSDs that are unsuitable for spray drying or HME [142]. A high-throughput miniaturized coprecipitation screening platform enables efficient selection of polymer type, drug loading, and solvent–antisolvent ratio, supporting both early and late-stage formulation development.

#### 5.2.5. Spray Drying

Spray drying is a solvent-evaporation technique used to prepare SDs, particularly ASDs, to enhance the solubility and dissolution of PWSDs. In this method, the drug and hydrophilic polymer (e.g., PVP, HPMC, PEG, or Soluplus^®^) solution is atomized into a hot drying chamber, where rapid solvent evaporation produces microparticles with the drug molecularly dispersed in an amorphous polymer matrix [143]. The technique offers advantages such as suitability for thermolabile drugs, uniform drug distribution, particle size control, scalability, and continuous processing. However, it has limitations, including the use of organic solvents, residual solvent concerns, equipment cost, potential low yield, and risk of recrystallization during storage. Its efficiency depends on formulation factors (drug and polymer properties, drug-to-polymer ratio, solvent choice), process parameters (temperature, feed rate, atomization conditions), and environmental factors such as humidity [144]. A Design of Experiments approach was applied to optimize spray-dried ternary ASDs of ibuprofen containing HPMCP-HP55 and Kollidon VA 64 [145]. Results showed that only formulations with a 1:4 API/excipient ratio were fully amorphous, while others remained crystalline. The ASD formulations significantly enhanced ibuprofen solubility due to its amorphous state and the presence of amphiphilic Kollidon VA 64. Computational fluid dynamics is widely used to model spray drying but is limited by high computational cost, the need for validation, and reduced accuracy in complex systems [143]. Although machine learning models can achieve high accuracy, their performance relies heavily on high-quality datasets, and they may have limited ability to predict the effects of novel formulation or process parameters on dried powder characteristics. To improve gefitinib solubility and achieve colon-targeted delivery, ternary SDs containing gefitinib and varying ratios of polymers were prepared using the spray drying technique [146]. Eudragit S100 was incorporated for colon targeting, while PVP and HPMC were used as solubility-enhancing carriers. Dissolution studies demonstrated significantly enhanced drug release from HPMC-based dispersions at pH 7.2 (up to 95% over 15 h), with minimal release at pH 1.2 and 6.5, indicating effective colon targeting. Cytotoxicity studies on Caco-2 cells revealed no additional inhibitory effect compared to the pure drug, suggesting formulation safety. An interesting study evaluated the effect of hydrogen bonding between drugs and PVA/VA copolymer on the stability of high drug-loaded spray-dried ASDs [147]. Results showed that hydrogen bonding significantly improved stability in moderately crystallizing drugs (e.g., nimesulide vs. fenofibrate), had a limited impact on rapidly crystallizing drugs (naproxen, caffeine). However, ASD could not be clearly assessed in slowly crystallizing drugs (indomethacin and miconazole) that remained amorphous.

#### 5.2.6. Kneading

SDs prepared by the kneading method involve blending the drug with a hydrophilic carrier using a minimal amount of solvent to form a paste. The mixture is kneaded to promote intimate mixing and partial molecular dispersion, followed by drying and milling to obtain the final product [148]. This technique improves drug dissolution primarily through enhanced wettability and reduced crystallinity or partial amorphization of the API. The kneading method offers several advantages, including low thermal stress (hence suitable for thermolabile drugs), minimal equipment, reduced solvent use, and suitability for early-stage formulation screening. However, it also has limitations such as potential batch-to-batch variability, incomplete amorphization, residual solvent or moisture retention after drying, and scale-up challenges. Recent literature highlights continued interest in solvent-minimized and mechanochemical approaches aligned with kneading principles, with emphasis on polymer selection, drug–polymer miscibility, and stability optimization to prevent recrystallization and maintain long-term performance of ASDs [149]. Efavirenz exhibits poor aqueous solubility that limits its absorption. Phase solubility studies with different carriers identified PVP K-30 as a suitable polymer for preparing SDs [150]. Although solvent-evaporated dispersions showed an amorphous state, they exhibited reduced dissolution rates. Kneaded dispersions (4:1 drug:polymer) and corresponding physical mixtures demonstrated enhanced dissolution and improved stability upon storage compared to the pure drug. Gliclazide, a BCS class II antidiabetic drug, was formulated as SDs to enhance its solubility [151]. SDs were prepared using carboxymethyl chitosan and PVP K-30 in different drug-to-carrier ratios (1:1, 1:3, and 1:5) via kneading and solvent-evaporation methods. Both carriers significantly improved solubility and dissolution compared to the pure drug. The drug:carrier (1:5) kneaded formulation increased solubility ~9-fold, while the same ratio by solvent-evaporated system achieved complete drug release within 30 min. Characterization studies confirmed drug–polymer compatibility, altered particle morphology, and reduced crystallinity. Table 2 presents studies carried out on ASD-based formulations.

### 5.3. Crystallization Inhibition Strategies

An optimal ASD generates a supersaturated drug state (spring effect) and maintains it by preventing precipitation (parachute effect) [158]. While binary systems may achieve both effects, ternary systems incorporating additional polymer are sometimes required to enhance precipitation inhibition and sustain drug supersaturation [159]. Surface crystallization of amorphous drugs occurs much faster than bulk crystallization due to the high molecular mobility at the surface, making it a key failure mechanism for ASDs [160]. Applying ultrathin coatings of metals or polymers significantly inhibits crystallization by reducing surface mobility through interfacial drug–polymer interactions, thereby improving physical stability [161]. Surface modification improves the physical stability of amorphous drugs by reducing surface molecular mobility. Coating indomethacin and nifedipine with gelatin or chitosan, as well as surface treatment via milling with carnauba wax or blending with Eudragit^®^ E PO 100, significantly inhibited crystallization through interfacial drug polymer interactions [162]. To address the physical instability of ASDs, a solvent-free atomic layer coating technique was applied to deposit an ultrathin aluminum oxide layer onto spray-dried ezetimibe ASDs [163]. The coating significantly improved powder properties and prevented crystallization for up to two years under accelerated conditions, enabling higher drug loading without compromising stability or drug release performance. A newly derived dimensionless precipitation parameter, supersaturation holding capacity, was used to select suitable polymers for the development of ASDs of nisoldipine [164]. PVP polymers demonstrated superior crystal growth inhibition and effectively stabilized the amorphous drug. Spray-dried PVP-based ASDs improved solubility, dissolution, physical stability, flow properties, and oral bioavailability compared to crystalline nisoldipine.

## 6. Cocrystallization

Cocrystals are crystalline, single-phase, multicomponent systems comprising two or more distinct molecular and/or ionic compounds that exist in a specific stoichiometric ratio and are mainly solids at room temperature [165]. Pharmaceutical cocrystals typically consist of API and pharmaceutically acceptable ingredients or coformers formed via noncovalent interactions such as hydrogen bonding, co-ordinate bonding, Van der Waals forces, or π-π stacking interactions [166]. A coformer possesses the capacity to adjust the physicochemical properties of the APIs through non-covalent interactions, all while leaving the pharmacological properties unchanged. These cocrystals provide a distinctive benefit by retaining the therapeutic effects of the API while improving the range of physicochemical characteristics, such as solubility, melting point, hygroscopicity, dissolution rate, stability, compressibility, permeability, tabletability, decreasing the bitter taste, bioavailability, and others [167]. Different theoretical crystal design approaches, such as hydrogen bonding propensity [168], supramolecular synthon [169], Cambridge Structure Database [170], pKa values [171], and Hansen solubility parameters [172], have been utilized for the selection of coformers. Cocrystals are categorized according to their composition, which includes the nature (such as ionic, polymorphic, hydrates, and solvates), as well as the number of components present in their crystal structure (for example, binary, ternary, and quaternary cocrystals).

Over the past few decades, cocrystallization has demonstrated its capability to enhance the in vivo performance of PWSDs by improving their solubility and bioavailability [173]. Ketoconazole has limited bioavailability due to its extremely low water solubility. However, the cocrystal of ketoconazole with p-aminobenzoic acid demonstrated a 10-fold increase in aqueous solubility and a 6.7-fold increase in oral bioavailability compared to crystalline ketoconazole [174]. The clinical utility of apigenin, a bioflavonoid, is constrained by its notably low solubility and bioavailability. Through the formation of a cocrystal with 4,40-bipyridine, the bioavailability of apigenin was significantly augmented (3.9-fold) compared to the unmodified drug [175]. Ambrisentan demonstrates poor water solubility (0.06 mg/mL), hence considered a BCS class II. The cocrystal of the drug with glycylglycine is prepared by the supramolecular synthon networks, the carbonyl in ambrisentan interacts with the OH acid groups of glycylglycine as well as the OH acid of another ambrisentan molecule, leading to the bifurcated acid interactions [176]. This cocrystal exhibited a 2.7-fold increase in Cmax and a nearly two-fold rise in AUC compared to the pure drug. In an effort to overcome the low aqueous solubility and bioavailability of oridonin, researchers successfully synthesized the drug-nicotinamide cocrystal [177]. Comparative studies reveal that the solubility and oral bioavailability of the cocrystal were enhanced by 1.34 and 1.18 times, respectively, compared to the unmodified drug. Cocrystals of berberine were prepared between the coformer (3-methylcinnamic acid) and the drug using the solvent-evaporation technique, which involves self-assembling through hydrogen bonds and π–π stacking interactions [178]. Compared to pure drug, the solubility of the non-hygroscopic cocrystal in polar solvents such as water, methanol, ethanol, and isopropanol was increased by 13.9, 1.5, 4.7, and 15.8 times, respectively. Additionally, the apparent absorption and absorption rate constants increased 7.7- and 5.6-fold, respectively. Flavonoids bearing a phenolic group at the 5-position can form intramolecular hydrogen bonds with the carbonyl group at the 4-position, a feature commonly observed in cocrystals of compounds such as hesperetin, genistein, and baicalein [179]. The coformers of flavonoid cocrystals also have some structural characteristics. Flavonoids tend to form cocrystals with N-containing heterocyclic compounds such as nicotinamide, isonicotinamide, theophylline, caffeine, 4,4′-bipyridine, proline, etc. An investigation was aimed at assessing the impact of various factors on cocrystal dissolution behavior, including the drug dose/solubility ratio (Do = Cdose/Sdrug), the solubility advantage of cocrystal over the drug (SA = Scocrystal/Sdrug), and the influence of dissolution media [180]. Solubility advantage value for 1:1 cocrystals of meloxicam-salicylic acid and meloxicam-maleic acid demonstrated significant enhancement, particularly in the pH range of 1.6 to 6.5. During dissolution, cocrystals regulated interfacial pH, influencing dissolution rate dependence on bulk pH. The study observed a range of Do values, mostly determined by drug solubility dependence on pH. When Do >> SA, dissolution-precipitation quasi-equilibrium state sustained supersaturation, while Do << SA indicated sufficient cocrystal solubility to dissolve the dose. Different cocrystal-to-drug conversion pathways were identified, shedding light on the dissolution–supersaturation–precipitation behavior. This interplay offers insights into understanding the behavior of cocrystals during dissolution.

It is worth mentioning that in the aqueous environment of the GIT, undesired solution-mediated phase transformations of cocrystals to more stable forms can hinder efficient drug delivery, thereby affecting the desired drug concentrations in circulation [181]. These phase transformation changes may involve different polymorphs, hydrated forms, or mixtures of the API and coformer. Various factors, including polymers, surfactants, biorelevant media, organic solvents, and pH, can significantly impact these physical changes. Weakly basic drugs experience significant decreases in solubility and dissolution as pH rises from one to seven along the GIT. Cocrystals of the basic drug ketoconazole with acidic coformers like adipic, fumaric, and succinic acids show a parabolic solubility trend with increasing pH, reaching a minimum before rising again [182]. These cocrystals exhibit pHmax values around 3.6 and 3.8, beyond which they cause supersaturation relative to the drug. The cocrystal supersaturation index varies significantly with pH changes from 1 (pHmax) to 10–30 (pH 5) to 800–3000 (pH 6.5), influencing cocrystal conversion during dissolution. Analyzing cocrystal dissolution behavior in terms of parameters like Cmax, σmax (maximum drug concentration and supersaturation), and AUCdiss (area under the curve of drug concentration during dissolution), reveals advantages in drug exposure for certain cocrystals. Understanding the interplay between supersaturation threshold, cocrystal solubility, and supersaturation index is crucial for optimizing cocrystal development, especially considering their pH-dependent behavior and potential implications for drug exposure.

### 6.1. Strategies for Cocrystal Design and Preparation

Numerous types of cocrystals have been conceived, developed, and analyzed utilizing various theoretical and experimental methods. These efforts have successfully enhanced the desired physicochemical properties of class II and class IV drugs, including solubility and permeability [183,184]. Different techniques have been reported for preparing cocrystals, including environmentally friendly solid-state grinding, solution reaction crystallization, solvent evaporation, slurry conversion, and HMEIn solution-based methods, ternary systems of API, coformer, and solvent are employed, with optimal conditions achieved when the cocrystal is supersaturated while the API and coformer remain saturated or undersaturated [185]. When components have significantly different solubilities, the less soluble one tends to precipitate first, leading to a solid mixture of cocrystal or unsuccessful cocrystal formation. An example of this is the synthesis of a block-shaped single crystal of febuxostat-piroxicam (1:1) cocrystal, achieved through slow evaporation of acetonitrile at room temperature over several days. The resulting cocrystal exhibited higher solubility and better tabletability compared to its individual components [186]. Similarly, cocrystals of nebivolol hydrochloride-nicotinamide with improved dissolution rates were obtained through solvent evaporation [187].

#### Thermodynamic Modeling and Predictive Approaches

The hard-chain-based perturbed-chain statistical associating fluid theory model was used to predict cocrystal solubility in solvent/antisolvent systems, improving formation efficiency. Moreover, this perturbation theory is a reliable in silico tool for predicting cocrystal stability under varying relative humidity by estimating deliquescence and phase behavior [188]. It also identifies the impact of impurities and excipients on stability, with predictions aligning well with experimental data to support formulation and storage decisions.

### 6.2. Methods of Cocrystal Preparation

#### 6.2.1. Supercritical Antisolvent

The supercritical antisolvent method has been investigated as a potential cocrystallization method for the formation of cocrystals (2:1) of the anti-inflammatory drug diflunisal and nicotinamide [189]. The dissolution rate of these supercritical antisolvent cocrystals was found to be higher than that of pure diflunisal. Furthermore, the cocrystals display identical crystal structures, thermal behaviors, and FTIR spectra similar to those of cocrystals prepared through liquid-assisted ball mill grinding and solution crystallization.

#### 6.2.2. Solution-Based Cocrystallization Methods

##### Cooling Crystallization

Cooling crystallization represents a commonly employed technique for scaling up production of purified cocrystals. The synthesis of a cocrystal involving fisetin, a bioactive natural hydrophobic flavonol known as 2-(3,4-dihydroxyphenyl)-3,7-dihydroxychromen-4-one, with caffeine, (1,3,7-trimethylpurine-2,6-dione), and nicotinamide (pyridine-3-carboxamide) co-formers, utilizing cooling crystallization technology, has been documented [190]. This approach aims to enhance the biopharmaceutical properties of fisetin, addressing limitations such as poor aqueous solubility and bioavailability. The cocrystal with caffeine exhibited a significant three-fold increase in the oral bioavailability of fisetin, while also maintaining its anti-inflammatory potential in the pharmacodynamic study.

##### Evaporative Cocrystallization

This method is a frequently employed technique for producing cocrystals, especially those intended for XRD studies, with a focus on generating single cocrystals. A cocrystal of carbamazepine with saccharin, prepared via solvent evaporation, demonstrated significantly improved dissolution behavior compared to the pure drug [191]. The enhancement in solubility is attributed to the modification of the crystal lattice and intermolecular interactions, which reduce lattice energy and improve wettability. Despite its simplicity and effectiveness, the method is generally limited by long processing times and challenges in large-scale production. However, it remains a valuable approach in early-stage formulation development and crystal engineering studies.

##### Reaction Cocrystallization Method (RCM)

RCM becomes a practical approach for cocrystal formation when the components of the cocrystal, with different solubilities, are combined to create supersaturated solutions, leading to the precipitation of the cocrystal [192]. This approach involves generating a solution supersaturated with respect to the cocrystal, while remaining saturated or undersaturated with respect to the individual components. Many investigations have showcased the application of RCM for synthesizing cocrystals, particularly focusing on drugs categorized under BCS class II. The encouraging outcomes achieved through RCM underscore its potential for producing pharmaceutical cocrystals that enhance the biopharmaceutical properties of drugs. The solubility of carbamazepine cocrystals with various coformers based on eutectic concentrations in water, ethanol, isopropanol, and ethyl acetate was studied. The findings revealed a significant increase, ranging from two to 152 times, in the solubility of cocrystals compared to the stable drug dihydrate form [193]. The cocrystal formed through RCM using 6-mercaptopurine and coformer isonicotinamide exhibited solubility enhancement ranging from 1.7 to 2.3 times greater, as well as a remarkable increase (168.7%) in bioavailability compared to the pure drug in three different buffers [194]. A study assessing the solubilization of seven distinct cocrystals acquired through RCM in biorelevant media has been described [195]. The findings indicated enhancements in solubility for the cocrystal in acetate buffer at pH 5 and simulated intestinal fluid in the fed state. The comparative solubility study of lamotrigine cocrystals and salts synthesized through RCM revealed that the drug–nicotinamide cocrystal exhibited the highest aqueous solubility compared to the hydrochloric acid salt, saccharin salt, and less soluble lamotrigine cocrystals with methylparaben and phenobarbital [196]. This solubility advantage was attributed to the interplay between the chemistry of both solid and solution phases.

##### Isothermal Slurry Method

It is a process of phase transformation mediated by solution, necessitating the addition of excess cocrystal components to the solvent. The ternary phase diagram directs the suggested range of concentration and temperature for the component, guiding the generation of cocrystal supersaturation. It was found that the rate of formation of the theophylline–benzoic acid cocrystal was notably influenced by the initial concentration of the components and the operating temperature, as assessed through in-line Raman spectroscopy [197]. However, incompatibility in the slurry state may arise due to differences in solubility and stability of the API–coformer system. For example, fluoxetine hydrochloride–succinic acid cocrystals have been reported to dissociate during slurry experiments in water, resulting in recrystallization of the parent API, while the coformer remained in solution due to its higher solubility [198]. This behavior reflects solution-mediated phase transformation and highlights the instability of certain cocrystals under slurry conditions. On the other hand, when thermodynamically stable conditions are achieved, slurry methods can yield phase-pure cocrystals. Studies have shown that a high proportion of slurry-generated cocrystals are devoid of residual starting materials, with up to 96% purity confirmed by powder X-ray diffraction analysis [199]. Similarly, systems such as theophylline–hydroxybenzoic acid have been successfully obtained as phase-pure cocrystals through slurry-based screening [200].

#### 6.2.3. Solid-State Methods

##### Solid-State Grinding

The dry and liquid-assisted grinding techniques are commonly employed for solid-state grinding to produce cocrystal powder samples. The API and coformers are combined in suitable stoichiometric ratios, then subjected to pressure and grinding using either a mortar and pestle, a ball mill, or a vibrator mill up to approximately 30–60 min. However, challenges associated with dry grinding may encompass difficulties in cocrystal formation, incomplete conversion to the desired cocrystal structure, and the emergence of crystalline defects, potentially leading to the presence of some amorphous content [201]. The liquid-assisted grinding process involves blending the two components by introducing a minute quantity of solvent, leading to accelerated kinetics of cocrystal formation. The benefits include enhanced efficiency, the capacity to control polymorph production, and the enhanced crystalline quality of the resulting product. Limitations include being a small-scale technique, requiring high energy consumption, and having low performance in terms of product purity.

#### 6.2.4. Thermal and Melt-Based Techniques

##### HME Method

In the HME method, cocrystals are prepared without the use of solvent by subjecting the drug and coformers to a specific temperature while intensively mixing them. The formation of cocrystals via the HME method necessitates the presence of a catalyzing agent to enhance cocrystal formation [202]. Additionally, HME matrices should have a lower Tg than the melting point of the cocrystal to enable processing at reduced temperatures. They should also exhibit limited noncovalent interactions with the drug or coformer and enable a swift solidification step. However, a drawback of this approach is that not all coformers and APIs are compatible in their liquefied states, rendering them unsuitable for use with unstable drugs. A representative example of incompatibility in the molten state can be observed in carbamazepine-based systems. Although carbamazepine forms several cocrystals with different coformers, studies have shown that during thermal processing, these systems may undergo polymorphic transformations or form amorphous phases instead of a stable cocrystal, particularly when molecular mobility is insufficient. For instance, melt growth of carbamazepine–nicotinamide cocrystals has been associated with the simultaneous formation of amorphous material alongside crystalline phases, indicating incomplete or unstable cocrystallization under thermal conditions [203]. Similarly, carbamazepine–saccharin cocrystals exhibit multiple polymorphic forms with temperature-dependent stability, where metastable forms may transform under thermal or processing conditions [204]. These observations suggest that even well-established cocrystal systems can display limited stability or incompatibility in the molten state, thereby posing challenges for successful cocrystal formation during HME processing.

##### Melting Crystallization

Melting crystallization offers an eco-friendly method for producing pharmaceutical cocrystals without utilizing any solvents. However, it is important to assess the thermal stability of both the drug and the coformer. The carbamazepine–nicotinamide cocrystal was synthesized by melting physical mixture of the drug and coformer at 160 °C, followed by gradual cooling to ambient temperature to facilitate crystal growth. The aqueous solubility of the prepared cocrystal shows no significant difference compared to the drug, while exhibiting a significant difference in solubility in ethanol. The findings of this study validate the significance of solvent composition and temperature as critical parameters in drug transformation within solution [205].

## 7. Solubilization Techniques

### 7.1. pH Modification

It is a frequently employed technique to enhance the solubility of PWSDs, especially weak acids and weak bases. The principle is based on the pH-dependent ionization of drugs, where the dissociated form of the drug is more soluble in water than the undissociated form. The relationship between pH, pKa, and the degree of dissociation of a drug is explained by the Henderson–Hasselbalch equation [206]. The advantages of pH adjustment include its simplicity, low cost, and rapid improvement of drug solubility without requiring complex equipment or processes. Examples of drugs utilizing pH adjustment include phenytoin sodium injection, which requires alkaline pH for solubility, lidocaine hydrochloride injection, which is formulated in acidic conditions, and ketoconazole, whose solubility increases in acidic environments. It is particularly useful in liquid formulations where the drug must remain completely dissolved. However, the technique applies only to ionizable drugs, and precipitation may occur when the formulation encounters physiological fluids with different pH after administration. In addition, extreme pH conditions may cause irritation, drug instability, or degradation, and maintaining the desired pH during storage may be challenging. Recent advancements in this technique involve combining pH-based solubility enhancement with modern drug delivery systems. Examples include pH-responsive nanoparticles and polymeric carriers that release drugs selectively in acidic tumor environments [207]. In the pH-modification approach, the microenvironmental pH around the drug particles at the diffusion site can be adjusted by incorporating pH-modifying excipients into the formulation [208]. This localized pH change can enhance the dissolution of drugs that exhibit pH-dependent solubility, which may ultimately improve oral absorption.

### 7.2. Co-Solvency

Co-solvency is a solubility enhancement technique in which water-miscible organic solvent (e.g., ethanol, propylene glycol, PEG 400, or glycerin) is added to water to reduce the polarity of the system, thereby improving the solvation and apparent solubility of hydrophobic drug molecules [209]. Most commonly used cosolvents are listed in Table 3. For instance, ethanol is a widely used pharmaceutical solvent due to its intermediate polarity, which enables dissolution of many PWSDs [210]. It is commonly used in elixirs, tinctures, injectable formulations, and in the extraction of bioactive compounds, contributing to drug efficacy, stability, and bioavailability. A co-solvent-based intravenous formulation of the poorly water-soluble isosteviol sodium was developed using ethanol (25%) and propylene glycol (20%) in saline at pH 10 [211]. The formulation remained stable for over three months, showed no significant hemolysis and good cytocompatibility, and demonstrated acceptable pharmacokinetic and safety profiles in rats, indicating improved solubility and potential for clinical application.

The major advantages of co-solvency include its simplicity, rapid formulation development, ease of scale-up, and the use of pharmaceutically accepted excipients. It is particularly useful in oral liquids, parenteral solutions, and topical formulations, and can also be combined with other approaches such as surfactants or pH adjustment. Appropriate selection of cosolvent systems is important, as it can enhance the solubility of PWSDs and support the development of improved oral formulations such as tablets and solutions. For instance, acyclovir, a poorly soluble and permeable antiviral drug, was investigated for solubility enhancement with particular emphasis on the cosolvency approach for oral dosage forms [224]. In the cosolvency study, six different binary solvent systems were evaluated at 298.15 K, demonstrating that solvent composition significantly influenced drug solubility. Among the systems tested, the PEG 400–water mixture (0.5:0.5) produced approximately a two-fold increase in solubility. However, co-solvency also has limitations, including the risk of drug precipitation upon dilution (e.g., after oral administration or intravenous infusion), potential toxicity or irritation associated with high cosolvent concentrations, and possible stability or compatibility issues with packaging materials. Moreover, for BCS class IV drugs, improvement in solubility alone may not sufficiently enhance bioavailability due to concurrent permeability limitations.

Drug solubility in cosolvent systems often follows a log-linear relationship with cosolvent fraction, which allows prediction and optimization during formulation development [225]. Early solubility modeling began with Hildebrand’s approach for nonpolar systems, and Yalkowsky’s general solubility equation further enabled the prediction of aqueous solubility using physicochemical parameters such as logP and melting point. Among these, the CNIBS/Redlich–Kister equation, later expanded as the Jouyban–Acree model, has provided some of the most accurate predictions across solvent compositions and temperatures, with ongoing research continuing to refine computational solubility modeling [226]. The log-linear cosolvency model was used to assess the solubility of ritonavir, griseofulvin, itraconazole, and ketoconazole in PVP using monomer–polymer solvent mixtures [227]. Solubility decreased in a log-linear manner as the system became more polymeric, with overall low solubility (<1–15% *w*/*w*). Ritonavir showed the highest solubility, while polymerization most adversely affected griseofulvin and itraconazole.

With a fixed number of independent variables, most cosolvency models show accuracy. However, their accuracy declines as more variables are included. To address this gap, generalized trained models have been developed to estimate drug solubility in binary solvent systems under isothermal and varying temperature conditions. Correlative models typically show 1–10% mean percentage deviation, whereas predictive models exhibit higher errors (10–80%), depending on model complexity and variables used [228]. Recent advancements in this area involve combined cosolvency approaches with hydrotropy, surfactants, and nanocarriers, as well as the development of green cosolvents and deep eutectic solvents (DESs), which improve solubilization efficiency while reducing toxicity [229].

The cosolvency behavior of S-ketoprofen was investigated in alcohol-water and acetone-water mixtures over the temperature range of 278.15–318.15 K [230]. Solubility increased with decreasing water content and showed a pronounced cosolvency effect in acetone–water at w = 0.80, where maximum solubility was observed. Hansen solubility parameters and preferential solvation analysis confirmed strong solute-cosolvent interactions, with hydrogen bonding and dipolar interactions playing dominant roles in dissolution. The solubility data were accurately correlated using Jouyban–Acree-based models, demonstrating their reliability in describing cosolvency systems. Cosolvency in amino acids was attributed to conformational changes and altered inter/intramolecular interactions. Molecular dynamics simulations effectively predicted its behavior and underlying mechanism [231]. Cosolvency was studied at the molecular level using tolbutamide as a model drug to understand its underlying mechanism. Experimental, spectroscopic, and simulation studies showed that changes in solute conformation, supramolecular clustering, and inter/intramolecular interactions drive the occurrence of cosolvency in different solvent systems [232]. Cosolvency was evaluated to enhance the solubility of sulfamethazine in acetonitrile-ethanol mixtures over 278.15–318.15 K [233]. Solubility increased linearly with acetonitrile fraction and temperature, indicating that acetonitrile is the more efficient cosolvent and that dissolution is endothermic and entropy-driven. The enhanced solubility was attributed to favorable solute–cosolvent interactions and lower energetic requirements for cavity formation, providing thermodynamic insight for formulation design. Cosolvent and PEG-based solubilization techniques are widely used to improve the delivery of PWSDs [209]. These approaches rely on appropriate excipients and physicochemical principles to enhance drug solubility. Cosolvents are often combined with surfactants to increase solubilization capacity and facilitate in vivo emulsification of self-emulsifying formulations. In PEG-based systems, drugs may be present as micronized crystalline particles (eutectic mixtures) or in amorphous form, which improves drug absorption due to faster dissolution and increased transient solubility in the GIT.

### 7.3. Hydrotropy and Mixed Solvency

#### 7.3.1. Mechanisms of Hydrotropic Solubilization

Hydrotropy, often described as the salting-in phenomenon, is a water-based solubility enhancement approach that increases the solubility of PWSDs without chemically modifying their molecular structure [234]. Hydrotropy represents a green, economical, and environmentally friendly alternative to conventional organic solvents, significantly enhancing the aqueous solubility, dissolution rate, and potential bioavailability of PWSDs. Furthermore, hydrotropic systems offer advantages including reduced toxicity, improved safety profiles, cost-effectiveness, compatibility with aqueous pharmaceutical processes, and broad applicability in pharmaceutical formulation and analytical methods [234]. This method involves the addition of high concentrations of hydrotropes, which are small, typically amphiphilic organic ions or molecules, such as sodium benzoate, sodium salicylate, nicotinamide, urea, and citrate. Key characteristics include the requirement for relatively high hydrotrope concentrations, often in the molar range, leading to the formation of non-micellar, dynamic drug–hydrotrope aggregates that are distinct from classic surfactant micelles. The effect can be further enhanced through mixed hydrotropy by using a combination of two or more hydrotropes to reduce the amount of any single agent required. Table 4 and Table 5 summarize various studies on hydrotropy-based solubility enhancement and recent patents on hydrotropy-based innovations, respectively. Limitations include the requirement for large excipient loads, which may increase osmolality and cause taste, irritation, or tolerability concerns depending on the route of administration. Additionally, potential precipitation upon dilution and formulation constraints, such as changes in viscosity, ionic strength, and excipient compatibility, are also documented. Moreover, hydrotropic agents may exhibit weak interactions with certain drugs, limiting solubilization efficiency. Since water is used as the primary solvent, its complete removal may not always be achievable. Furthermore, the use of some hydrotropes is restricted due to potential toxicity concerns.

Hydrotropic solubilization has been explained by three main mechanisms: complex formation, self-aggregation of hydrotropes, and modification of water structure. The spontaneous aggregation theory suggests that hydrotrope molecules self-associate in aqueous media to form organized, non-micellar clusters once a threshold concentration, known as the minimum hydrotropic concentration, is reached [243]. These aggregates, often formed through stacking interactions of planar aromatic rings, create structured microenvironments that can entrap hydrophobic solute molecules, thereby enhancing their apparent solubility. This aggregation process is accompanied by changes in thermodynamic parameters such as enthalpy, entropy, and free energy, which collectively facilitate drug solubilization [244]. The complexation theory proposes that solubilization occurs through weak and reversible interactions between the drug and hydrotrope molecules [245]. These interactions may involve self-association or hetero-association, often driven by π-π stacking between planar structures to minimize exposure to water. Complexes may form in a 1:1 ratio or a 1:2 ratio, where two hydrotrope molecules surround the drug molecule to form a sandwich-type complex, further enhancing aqueous solubility. The water-structure modification theory suggests that hydrotropes disrupt the tetrahedral hydrogen-bonded network of water. By reducing the surface tension of water and destabilizing structured forms such as ice-like clusters, hydrotropes weaken the barrier between solute and solvent. This reduction in interfacial tension enhances the dispersibility of weakly soluble solutes and promotes their dissolution in aqueous media [246].

Studies demonstrate that hydrotropic agents such as sodium benzoate, sodium salicylate, and urea effectively enhance solubility and allow accurate, eco-friendly, and cost-effective analysis without organic solvents [247]. Hydrotropic solubilization has been widely applied in titrimetric and spectrophotometric analysis to enhance the aqueous solubility of PWSDs, eliminating the need for volatile and toxic organic solvents. Hydrotropy was applied to enhance the dissolution and oral efficacy of poorly water-soluble ebastine through co-processing with caffeine, which acted as both a hydrotrope and a cocrystal former [248]. Caffeine significantly improved drug dissolution, particularly at higher ratios due to its hydrotropic solubilizing effect, leading to enhanced in vivo anti-inflammatory activity compared to pure ebastine.

#### 7.3.2. Mixed Hydrotropy

It is another solubility enhancement technique that uses a combination of two or more hydrotropic agents at lower individual concentrations to achieve synergistic improvement in the aqueous solubility of PWSDs [249]. It enhances solubility through combined intermolecular interactions such as hydrogen bonding and ionic interactions, offering a cost-effective, eco-friendly alternative to organic solvents while improving dissolution and potential bioavailability. Nevirapine, a BCS class II antiretroviral drug with dissolution-limited absorption, was formulated using a mixed hydrotropy approach to enhance its solubility and dissolution rate [250]. Screening of individual and combined hydrotropic agents identified lactose-citric acid blend (15:25, total 40%) as optimal, which was used to prepare SDs showing improved solubility without drug–hydrotrope incompatibility.

#### 7.3.3. Mixed Solvency

It is another solubility enhancement approach that uses a blend of multiple water-miscible solubilizers (e.g., hydrotropes, co-solvents, buffers, salts) at individually low, safer levels to achieve additive/synergistic increases in the aqueous solubility and dissolution of PWSDs. This strategy aligns with green/white chemistry by minimizing toxic organic solvents and has been applied across formulation development (e.g., SDs, liquids) and various pharmaceutical analyses, often improving performance without compromising practicality [251].

### 7.4. Micellar Solubilization

Micellar solubilization is an efficient technique used to improve the aqueous solubility of PWSDs by employing surfactants that form micelles in aqueous media above their critical micelle concentration. These micelles possess a hydrophobic core and a hydrophilic outer shell, allowing lipophilic drug molecules to be incorporated into the core or interfacial region, thereby increasing their apparent solubility and dissolution rate [252]. This approach offers several advantages, including improved drug solubility, enhanced dissolution and bioavailability, protection of drugs from degradation, and relatively simple and cost-effective formulation. Common surfactants used for micellar solubilization include non-ionic surfactants like polysorbates (Tween 80), Cremophor EL, and PEG derivatives, as well as ionic surfactants like sodium dodecyl sulfate, sodium dodecylbenzene sulfonate, lauryl macroglycerides, cetyltrimethylammonium bromide, and mono and di-fatty acid esters of low-molecular-weight PEGs. Using this approach, the solubility of several BCS class II antidiabetic drugs, including glyburide, glimepiride, gliclazide, glipizide, pioglitazone, repaglinide, and rosiglitazone, has been significantly enhanced through micellar solubilization [253]. However, the technique also has certain limitations, such as potential toxicity or irritation due to high surfactant concentrations and the possibility of drug precipitation upon dilution below the micellar concentration after administration. Recent progress in this area includes the development of polymeric micelles, mixed micellar systems, and stimuli-responsive micelles, which provide improved stability, higher drug loading capacity, and better targeted delivery of PWSDs [254].

### 7.5. Complexation Techniques

Among various solubility enhancement strategies, complexation is a widely employed and effective approach. Complexation involves the reversible interaction between a drug molecule (guest) and a complexing agent (host), resulting in the formation of a soluble molecular complex that improves the apparent solubility and dissolution rate of the drug [255]. The underlying principle of complexation is based on non-covalent interactions such as hydrogen bonding, van der Waals forces, hydrophobic interactions, and electrostatic forces. The most commonly used complexing agents in pharmaceutical systems are CDs, which possess a hydrophobic cavity capable of encapsulating lipophilic drug molecules while maintaining a hydrophilic exterior that enhances aqueous solubility. Other complexation systems include metal ion complexes, drug-organic molecule complexes, ion-exchange resins, and pharmacosomes [256]. The formation of these complexes shifts the equilibrium toward increased dissolved drug concentration without chemically modifying the drug molecule. However, careful selection of complexing agents and optimization of formulation parameters are essential to maximize its therapeutic potential. Complexation effects often depend on the inclusion method and the addition of ternary agents or co-components, which can further increase solubilization [257].

#### 7.5.1. Cyclodextrin-Based Systems

They are cyclic oligosaccharides composed of α-1,4-linked glucose units, produced through the enzymatic conversion of starch. The interior cavity of CDs is relatively hydrophobic due to the presence of CH_2_ groups, whereas the hydrophilic nature at the rim of the cavity is attributed to the primary and secondary hydroxyl groups located at its entrances [258]. This distinctive arrangement enables them to form inclusion complexes with hydrophobic drug molecules. Only molecules with a suitable size and stereochemistry can fit into the CD cavity through hydrophobic interactions. Among the different types, β-CD and γ-CD are the most widely used in pharmaceutical formulations due to their relatively larger hydrophobic cavity diameters (approximately 6 Å and 8 Å, respectively), which facilitate effective drug inclusion [255]. Determination of the association (stability) constant and complexation efficiency is crucial for CD complexes because these parameters directly influence formulation performance and feasibility [259]. One of the most frequently used methods of determining association constant and stoichiometric ratio is the phase-solubility technique [260]. The stability constant of CD complexes typically ranges from 0 to 100,000 M^−1^. Binding strength can be interpreted as follows: 0 indicates no binding; <500 M^−1^ reflects very weak binding; 500–1000 M^−1^ indicates weak binding; 1000–5000 M^−1^ corresponds to moderate binding; 5000–20,000 M^−1^ suggests strong binding; and values >20,000 M^−1^ represent very strong binding [261].

Complexation with CDs and their derivatives offers several advantages, particularly in drug delivery applications [262]. It improves aqueous solubility and dissolution rate of BCS class II and IV drugs without altering the pharmacological activity of the drug, enhances chemical and physical stability, reduces irritation and unpleasant taste, and can improve bioavailability. CD-based complexes are particularly valuable in oral, parenteral, ophthalmic, and topical formulations due to their safety profile and regulatory acceptance [263]. Native CDs such as α-CD, β-CD, and γ-CD form inclusion complexes with hydrophobic drug molecules, improving their aqueous solubility. Among them, β-CD is the most commonly used. However, its relatively low solubility has led to the development of modified derivatives such as HP-β-CD, sulfobutylether-β-CD (SBE-β-CD), methyl-β-CD (Mβ-CD), and carboxymethyl-β-CD, which provide significantly higher aqueous solubility and improved drug delivery performance [260]. Modified water-soluble β-CD derivatives are chemically altered forms of β-CD designed to improve aqueous solubility, enhance drug inclusion capacity, and increase their effectiveness as carriers in drug delivery systems. A newly modified water-soluble β-CD-epichlorohydrin derivative was investigated as a drug carrier to improve the solubility and bioavailability of galangin [264].

HP-β-CD showed greater affinity for sorafenib than SBE-β-CD, significantly enhancing its solubility [265]. CD complexation also improved intestinal permeability in EpiIntestinal^®^ models and increased cytotoxicity against various cell lines. These findings suggest that HP-β-CD can improve the bioavailability and therapeutic efficacy of sorafenib. The potential of inclusion complex (1:1) to enhance the efficacy of acyclovir using HP-β-CD was demonstrated by higher dissolution efficiency and greater bioavailability [266].

Many phytochemicals exhibit poor aqueous solubility due to their hydrophobic nature. This restricts their therapeutic effectiveness and necessitates solubility-enhancing strategies such as nanoformulations or CD complexation [14]. Phytochemicals such as flavonoids, phenolics, and triterpenoids show improved solubility, dissolution behavior, stability, and biological activity when complexed with CDs. Complexation of resveratrol with SBE-β-CD at a 1:1 ratio significantly improved its aqueous solubility and stability, resulting in prolonged half-life at physiological pH and in plasma [267]. The formulated complex exhibited suitable aerodynamic properties for pulmonary delivery and maintained its cytotoxic activity against non-small-cell lung cancer cell lines, indicating its potential as an inhalable dosage form. Recent investigations in drug-CD and phytochemicals-CD are summarized in Table 6 and Table 7, respectively.

##### Factors Influencing Complexation

The formation and stability of CD inclusion complexes depend on factors such as drug-CD molecular structure, cavity size, charge, degree of substitution, and environmental conditions, including pH, solvent, and temperature [292,293]. Molecular size compatibility and lipophilicity are critical; for example, poorly water-soluble drugs such as ibuprofen (R- and S-forms) form more stable complexes with β-CD due to better cavity fit [294], while moderately lipophilic drugs like naproxen show optimal inclusion in β-CD [295]. Substituted CDs, such as HP-β-CD, enhance the solubility of itraconazole [296], and the anionically charged SBE-β-CD improves the solubility of cationic drugs like prazosin and papaverine through combined inclusion and electrostatic interactions [297]. Environmental factors such as pH and temperature can significantly influence the stability constant of ionizable guest molecules, such as ibuprofen and bisdemethoxycurcumin, respectively [298]. Additives such as polymers, such as PVP, can further enhance complexation, as reported for pimozide [299]. Freeze-drying enhances the formation of well-defined CD inclusion complexes by promoting crystallization during freezing, often producing channel-type structures with guest molecules (e.g., benzoic acid derivatives and aspirin). In contrast, conventional coprecipitation may yield different crystal forms or fail to form complexes, demonstrating the superior efficiency of freeze-drying [300]. CD complexation combined with salt formation significantly enhances the solubility and performance of acidic drugs [301]. In the case of diclofenac, the sodium salt form exhibits markedly higher aqueous solubility (>10 mg/mL at pH 5–7) when complexed with CDs compared to the poorly soluble free acid form. Finally, complexation efficiency can be further enhanced by forming a water-soluble derivative (e.g., benzodiazepine derivatives) of a poorly water-soluble drug, possessing higher intrinsic solubility than the parent compound [302].

An important practical consideration is formulation bulk, as CDs have relatively high molecular weights and may substantially increase the final dosage size [255]. The extent of bulk increase depends on the drug-to-CD ratio and complexation efficiency. Drugs with low potency or low complexation efficiency require larger amounts of CD, potentially exceeding acceptable oral dosage limits (generally ~800 mg per unit). Consequently, CD complexation is more suitable for potent or moderately potent drugs with favorable CE values. Additionally, solvent selection (preferably water), excipients, processing methods (e.g., spray drying or freeze drying), and temperature must be optimized, as they directly influence complex stability, solubility enhancement, and overall formulation feasibility. Complexation efficiency of drug-CD systems can be enhanced by strategies that increase drug solubility or promote stronger host–guest interactions, such as drug ionization, salt formation, amorphization, ion pairing, and the use of co-solvents. The incorporation of water-soluble polymers, hydroxy acids, or metal ions may further improve CE through ternary or multicomponent complex formation. In practice, combining two or more of these approaches often produces a synergistic effect, leading to greater solubility enhancement and improved formulation performance [303]. In some cases, the complex may dissociate upon dilution in biological fluids, affecting in vivo performance. Additionally, not all drugs are structurally suitable for effective inclusion complex formation, and toxicity concerns may arise with certain complexing agents or at high doses.

##### Binary and Ternary Complexes

In a binary complex system, the hydrophobic portion of the drug/phytochemical molecule is partially or completely encapsulated within the hydrophobic cavity of the CD. A supramolecular ternary complex involves three components, usually a drug, CD, and an auxiliary substance such as a hydrophilic polymer (e.g., poloxamer) or an amino acid. In ternary complexes, the drug first forms an inclusion complex with CD. A third component, such as a polymer or co-complexing agent, then interacts with this binary complex to stabilize it. Ternary complexes exhibit higher complexation efficiency and stability than binary complexes and require lower CD concentrations to achieve optimal solubility and stability [304]. Recently, in silico molecular modeling and machine learning have emerged as important preliminary tools for evaluating both binary and ternary CD complexes. A machine learning model built from 596 ternary CD formulation datasets showed strong predictive performance (R^2^ = 0.887 for S_T_ (solubility of ternary CD complexes) and 0.815 for S_T_/S_B_ (solubility of binary CD complexes) [304]. Validation with hydrocortisone/β-CD/HPMC and dovitinib/γ-CD/carboxymethyl cellulose systems confirmed the reliability of the model. Molecular modeling further indicated that HPMC enhances solubility by preventing CD self-aggregation.

### 7.6. Advanced Cyclodextrin Systems

#### 7.6.1. Integration of Cyclodextrins with Metal–Organic Frameworks

Integration of metal–organic frameworks (MOFs) is a crystalline supramolecular material composed of metal nodes linked by organic ligands, resulting in highly porous structures [305]. The integration of β-CD-based metal–organic frameworks with polymers produced hybrid materials with superior properties compared to individual polymers or supramolecular matrices [306]. Two structures were developed, namely necklace-shaped, formed through potassium-mediated crystal growth and bridging, and dendritic sandwich-like, stabilized by hydrogen bonding and coordination interactions. Using curcumin as a model drug, both hybrids enhanced drug stability and demonstrated potential for controlled drug release, highlighting the promise of prepared hybrids for enhanced solubility and drug delivery applications. Among CD derivatives, γ-CD is most widely utilized due to its favorable structural features, enabling the formation of well-defined cubic three-dimensional frameworks with uniform amphiphilic nanopores [307]. γ-CD-based MOFs prepared with metal ions such as K^+^, Na^+^, and Fe^3+^ exhibited uniform mesoporous structures, good biocompatibility, and sustained drug release nearly~62% in 12 h [308]. These systems were successfully used to encapsulate anti-inflammatory drugs such as diclofenac sodium, showing controlled release and significant in vivo anti-inflammatory activity, with faster onset for K^+^/Na^+^ systems and prolonged effect for Fe^3+^ systems. These properties highlight their potential as efficient drug delivery carriers. Functionalization of CDs with moieties such as PEG, tetraethylene glycol, or mannose further improves immune evasion and targeting potential [309]. These modified CDs can be integrated onto MOFs (e.g., MIL-100(Fe) via phosphate–Fe^3+^ interactions), resulting in reduced macrophage uptake and improved drug delivery performance [310,311].

#### 7.6.2. Cyclodextrin–Hydrogel Hybrid System

**Figure 5:**
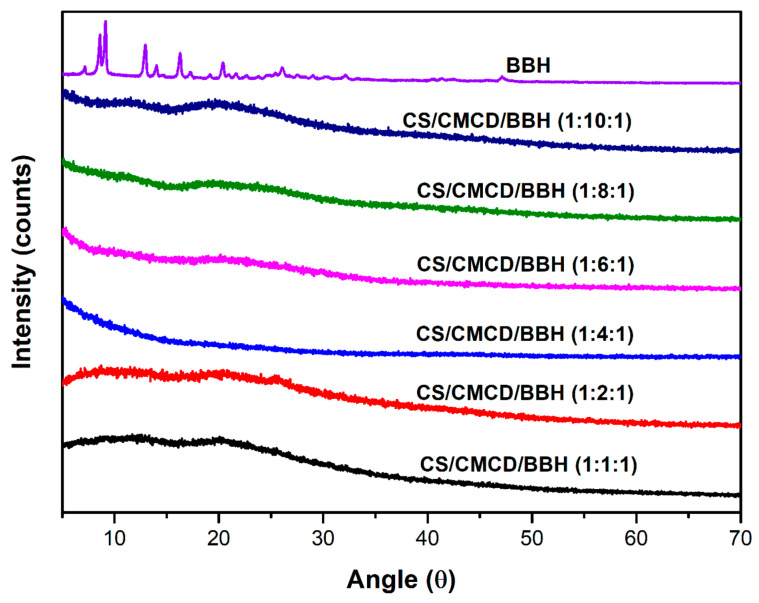
X-ray diffraction (XRD) patterns of berberine hydrochloride (BBH) and chitosan (CS)/carboxymethyl-β-CD (CMCD)/BBH hydrogels prepared with varying CMCD formulations. Reproduced from Ref. [313].

CD hydrogel hybrids are versatile drug delivery systems that enhance drug solubility, enable controlled release, and improve bioavailability [312]. A chitosan and carboxymethyl-β-CD-based oral hydrogel containing berberine hydrochloride was developed through physical cross-linking [313]. XRD analysis demonstrated a transition from crystalline to amorphous structure upon hydrogel formation and drug incorporation (Figure 5). While chitosan (at ~10–11° and 19–22°) and carboxymethyl-β-CD at 15–30°) exhibited characteristic crystalline peaks, the chitosan/carboxymethyl-β-CD hydrogel showed broadened peaks, indicating reduced crystallinity and increased amorphization. Similarly, berberine hydrochloride displayed distinct crystalline peaks (8.6°, 9.1°, 12.9°, 16.2°, 20.9°, 25.4°, 30.5°, 45.3°), which were diminished or broadened after incorporation into the hydrogel, confirming its amorphous dispersion.

## 8. Deep Eutectic Systems and Emerging Solvent Platforms

### 8.1. Deep Eutectic Solvents (DESs)

DESs are formed by combining hydrogen bond acceptor and donor in stoichiometric ratios, producing mixtures with melting points lower than their individual components [314]. DES systems such as urea-PEG mixtures may exhibit cosolvent-like behavior in aqueous media. However, they are mechanistically distinct from conventional cosolvency and are more appropriately classified as DES systems. The solubility of griseofulvin was investigated in aqueous DES systems composed of choline chloride/urea and choline chloride/PEG-300 over 298.15–313.15 K [315]. Drug solubility increased with both DES concentration and temperature, with choline chloride/PEG DES showing superior solubilizing ability and achieving solubility of 18.46 mmol·L^−1^ at 298.15 K. Spectroscopic, Hansen solubility parameter, density, and thermodynamic analyses confirmed favorable drug-DES interactions and highlighted the strong potential of polymeric DES as effective solubilizers.

### 8.2. Therapeutic Deep Eutectic Systems (THEDES)

THEDES enhance drug solubility, permeability, and bioavailability by combining drugs with eco-friendly DESs [316]. These biodegradable, low-toxicity solvents show strong potential in drug delivery and pharmaceutical development. A DES is generally composed of two components (hydrogen bond donor and acceptor) that interact through strong intermolecular hydrogen bonding, resulting in a significant reduction in the melting point of the mixture. Typically, the acceptor is a quaternary ammonium salt such as choline chloride, while the donor may include compounds like urea, glycerol, sugars, or organic acids. A classic example is the choline chloride-urea system, where hydrogen bonding between the chloride ion and hydrogen-donating groups disrupts the crystalline lattice of the individual components, leading to the formation of a stable liquid near room temperature. Fabrication methods for THEDES include electrospinning, self-emulsification, supercritical fluid processing, and nanoprecipitation techniques. THEDES are versatile drug delivery systems under investigation for multiple administration routes, including transdermal, oral, intravenous, buccal, and nasal, to improve drug bioavailability. Ionic liquids and DESs encounter significant challenges related to biocompatibility, the possible effects of impurities, and the need for a deeper understanding of their microscopic interactions. Gaining insight into these molecular-level interactions is essential for the rational design of task-specific solvents [317].

## 9. Nanotechnology-Driven Approaches

### 9.1. Lipid-Based Drug Delivery Systems

Another widely explored strategy involves lipid-based drug delivery systems, which enhance drug solubilization in the gastrointestinal environment and promote lymphatic transport. Lipid-based carriers improve drug absorption by maintaining the drug in a solubilized state during gastrointestinal transit. Liposomes are phospholipid vesicles that can encapsulate both hydrophilic and hydrophobic drugs, improving drug solubility and enabling targeted delivery [318]. Polymeric micelles, formed from amphiphilic molecules, possess a hydrophobic core that can solubilize PWSDs while their hydrophilic shell stabilizes the system in aqueous media [319]. Solid lipid nanoparticles (SLNs) consist of physiologically compatible solid lipid matrices that provide improved drug stability, controlled release, and enhanced bioavailability, making them attractive carriers for BCS class II and IV drugs [14,320]. Ivacaftor-loaded SLNs were prepared using homogenization and ultrasonication, employing Labrasol as the liquid lipid, cetyl palmitate as the solid lipid, and polysorbate 20 as the surfactant [321]. The optimized formulation demonstrated improved drug release, following a first-order kinetic model with sustained release from the lipid matrix. Nanostructured lipid carriers (NLCs) are advanced lipid-based drug delivery systems composed of a mixture of solid and liquid lipids that enhance drug loading capacity, stability, and controlled release of PWSDs. In vivo pharmacokinetic studies of 5-fluorouracil-based NLC in Wistar rats demonstrated enhanced activity of 5-FU by approximately 1.5-fold compared to the free drug [322]. This improvement is attributed to increased drug solubility and sustained release, leading to better bioavailability and therapeutic efficacy.

Nanoemulsion formulation refers to the development of a stable colloidal dispersion of oil and water stabilized by surfactants, where the droplet size typically ranges from 20 to 200 nm [323]. Such formulations are designed to improve the solubility, stability, and bioavailability of PWSDs, making them suitable for oral, topical, ocular, or parenteral drug delivery. A nanoemulsion formulation of azilsartan medoxomil was developed using ethyl oleate, Tween 80, and Transcutol P as the oil, surfactant, and co-surfactant, respectively [324]. The formulation significantly improved drug performance, demonstrating 1.71-fold higher drug release (enhanced solubility) and 2.1-fold greater intestinal permeation compared with the drug suspension.

Self-emulsifying drug delivery systems (SEDDS) represent another important approach for improving the oral bioavailability of PWSDs. SEDDS are isotropic mixtures of oils, surfactants, and cosolvents that spontaneously form fine oil-in-water emulsions when exposed to gastrointestinal fluids with gentle agitation [325]. The resulting micro or nanoemulsion droplets significantly increase the interfacial surface area, thereby improving drug dissolution and absorption [326]. In addition, SEDDS can enhance permeability by facilitating drug transport through intestinal membranes and protecting drugs from enzymatic degradation. For instance, sesamin is a PWSD that undergoes rapid hepatic metabolism, resulting in low oral bioavailability [327]. Formulation as a self-nanoemulsifying system significantly enhanced the performance of sesamin, increasing intestinal permeability by more than three-fold, relative bioavailability by approximately 12.9-fold, and absolute bioavailability from 0.3% to 4.4%. Zeta-potential-changing SEDDS represents a promising strategy because their small droplet size and ability to alter surface charge enable efficient diffusion through mucus [328]. Combining the zeta-potential-modulating approach with SEDDS can therefore enhance mucus permeation and drug diffusion, improving overall drug delivery efficiency.

### 9.2. Nanocarrier-Based Solubility Enhancement

Nanodrug delivery systems have emerged as one of the most effective approaches for improving the performance of PWSDs [329]. These systems reduce the drug particle size to the nanometer scale, thereby increasing the surface area and dissolution rate according to the Noyes–Whitney equation. Various nanocarrier systems employed for solubility enhancement are summarized in Table 8.

#### 9.2.1. Nanosuspensions

They consist of pure drug particles stabilized by surfactants or polymers and have been widely used to enhance the dissolution and bioavailability of PWSDs. The reduction in particle size also increases saturation solubility as explained by the classical Ostwald–Freundlich equation and the Gibbs–Thomson effect and enhances drug–membrane interactions, potentially improving permeability [339]. The nanosuspension technique is a practical approach for drug delivery when therapeutic molecules exhibit limitations such as poor aqueous solubility, high dose requirements, inability to form salts, large molecular weight, high log *p* values, or high melting points. This method helps overcome these challenges and improve drug performance [20]. For BCS class II drugs, such as griseofulvin, nanosuspensions enhance dissolution-limited absorption by increasing the particle surface area and improving interaction with gastrointestinal fluids [340]. According to the developability classification system, this approach is especially beneficial for class IIa drugs such as carbamazepine, where absorption is mainly limited by the dissolution rate [341]. Nanosuspension can also maintain drugs in a supersaturated state and prevent precipitation, helping to ensure consistent bioavailability. This strategy benefits drugs with pH-dependent solubility, such as indomethacin and verapamil, by maintaining solubility across varying gastrointestinal conditions.

#### 9.2.2. Polymeric Nanoparticles

They are colloidal carrier systems composed of biodegradable or biocompatible polymers (10–1000 nm) that encapsulate, adsorb, or solubilize APIs to enhance stability, solubility, and delivery. These systems improve the solubility of PWSDs and phytochemicals by reducing particle size, enhancing dispersion, and enabling controlled release, thereby increasing their bioavailability and therapeutic efficacy [331]. Lipid–polymer hybrid nanoparticles are advanced drug delivery systems that combine the structural integrity of polymeric cores with the biocompatibility and solubilizing properties of lipid shells. They enhance the solubility of PWSDs and phytochemicals by improving drug encapsulation, increasing wettability, and facilitating controlled release, thereby improving bioavailability [342].

#### 9.2.3. Nanosponges

Natural and inorganic nanosponges are promising carriers for PWSDs. Their porous, three-dimensional structure enables encapsulation of both hydrophilic and lipophilic molecules, enhancing solubility, providing controlled release, and supporting targeted drug delivery [343]. Ellagic acid, a polyphenolic compound with diverse pharmacological activities, is limited by poor solubility, stability, gastrointestinal permeability, and extensive first-pass metabolism. To overcome these limitations, the drug was encapsulated in CD nanosponges prepared by melt and microwave-assisted methods [344]. The optimized formulation showed satisfactory physicochemical properties, a 10-fold increase in solubility, enhanced photostability, and improved antioxidant activity, with the melt method demonstrating superior overall performance.

#### 9.2.4. Hybrid Cyclodextrin-Based Nanocarrier Systems

Hybrid CD nanocarrier systems are used when PWSD first benefits from CD inclusion complexation and is then incorporated into a nanocarrier to further improve apparent solubility, dissolution, loading, protection from precipitation/degradation, and, in many cases, oral or local bioavailability. Recent studies on nanocarrier systems combined with CDs for PWSDs are listed in Table 9. In liposomal systems, CDs first form inclusion complexes with hydrophobic drugs, which are then encapsulated in the aqueous core, enhancing drug loading and reducing leakage from the lipid bilayer [345]. Cross-linked CD nanosponges provide both inclusion and non-inclusion domains, enabling efficient encapsulation of PWSDs. Microwave-synthesized CD nanosponges ensure rapid and uniform heating [346]. This carrier was utilized for the dual loading of cinnarizine and domperidone to enhance their solubility and dissolution [347]. In vivo studies demonstrated significantly increased bioavailability (4.54-fold for cinnarizine and 2.90-fold for domperidone), highlighting the potential of microwave-based CD nanosponges for simultaneous multi-drug delivery. In lipid-based systems such as SLNs and NLCs, pre-complexation of drugs with CDs improves drug dispersibility, encapsulation efficiency, and modulates release behavior [348]. CDs are also integrated into emulsions and self-nanoemulsifying drug delivery systems, where drug–CD inclusion complexes can reduce burst release and improve formulation stability [349]. Furthermore, CDs are widely used in advanced nanocarriers, including mesoporous silica nanoparticles, CD-based metal–organic framework [350], nanogels [351], polymeric micelles [352], and nanofibers [353]. In these systems, the host–guest inclusion capability of CDs enhances drug loading, maintains hydrophobic drugs in a dispersed state, and enables controlled or stimuli-responsive drug release. Collectively, CD-based hybrid nanocarriers represent a versatile strategy to improve the solubility, stability, and therapeutic performance of PWSDs. This novel hybrid strategy combines the molecular solubilization effect of CDs with the carrier-level advantages of nanosystems such as liposomes, nanosponges, nanogels, emulsions, nanospheres, and lipid nanoparticles [354].

## 10. Formulation-Based Enabling Technologies

### 10.1. Liquisolid Systems

The liquisolid technique involves dissolving or dispersing PWSDs in non-volatile solvents and subsequently converting the liquid drugs into dry, free-flowing, and compressible powder using suitable carrier and coating materials [364]. Liquisolid systems increase drug wettability and surface area, resulting in faster dissolution rates and improved drug release from solid dosage forms. The improved dissolution behavior ultimately leads to enhanced bioavailability for drugs with poor solubility. The liquisolid compact technique was investigated to overcome the low aqueous solubility of carvedilol, which limits its oral clinical efficacy. Using PEG 400 (non-volatile solvent), Neusilin US2 (carrier material), and Aerosil 200 (coating material), the optimized formulation converted carvedilol into an amorphous form, resulting in improved dissolution (95% drug release in 30 min) [365]. Pharmacokinetic studies in rats further demonstrated enhanced absorption and increased systemic bioavailability, confirming that the liquisolid compact approach effectively improves the solubility and oral bioavailability of carvedilol. Additionally, formulation strategies like effervescent tablets have also been reported as an effective approach to improve dissolution of APIs [366,367]. Alternative approaches, like orodispersible systems as well as buccal films, are also explored to enhance the solubility of BCS class II drugs [368,369].

### 10.2. Use of Hydrophilic Polymers

**Figure 6:**
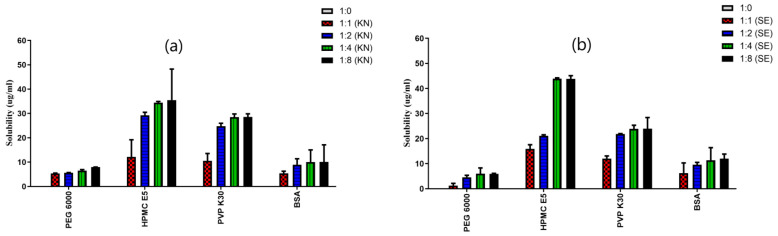
Solubility profiles of CUR, CUR:PEG 6000, CUR:HPMC E5, CUR:PVP K30, and CUR:BSA prepared at different phytochemical: polymer ratios (1:0,1:2,1:4, and 1:8) using the kneading method (KN) (**a**) and the solvent-evaporation method (SE) (**b**). Reproduced from Ref. [371].

Hydrophilic polymers are widely used as carriers to enhance the solubility and dissolution rate of hydrophobic drugs by improving stability and modifying drug release behavior. However, although many polymers are effective, the overall performance of SD largely depends on the specific carrier selected [370]. The effectiveness of hydrophilic polymers in enhancing the solubility and dissolution of poorly water-soluble curcumin was evaluated using SD techniques, including kneading and solvent-evaporation methods [371]. Dispersions were prepared with PEG 6000, HPMC E5, PVP K-30, and bovine serum albumin and characterized by solubility studies, dissolution testing, and material characterization. Among the formulations, HPMC E5 and PVP-based systems significantly increased solubility (4.3- and 2.8-fold, respectively), with HPMC E5 demonstrating superior morphological and solid-state modifications, confirming its suitability for developing stable ASDs of curcumin (Figure 6). A suitable carrier for the SD can be selected from a pool of candidates using polarized microscopy screening. Investigations revealed that the solubility and dissolution of poorly water-soluble etoricoxib were enhanced through the development of kneaded SDs using the most suitable carrier, Poloxamer 407 [372]. Compared with the pure drug and its physical mixtures, the SDs significantly improved performance, increasing solubility from 99.08 to 296.8 μg/mL and dissolution from 69.32% to 98.07%. The kneading method proved effective in producing ASDs with the potential to enhance bioavailability.

## 11. Future Perspectives and Challenges

Future research on PWSDs, including BCS class II and IV drugs and phytochemicals, should move beyond conventional solubility enhancement strategies toward integrated, mechanistically driven approaches that address both dissolution and absorption barriers. The design of multifunctional delivery systems, such as hybrid nanocarriers combining lipid-based systems with polymers, CDs, or hydrotropic agents, offers significant potential [342,373]. However, their clinical translation requires a deeper understanding of drug-excipient and carrier-biological interface interactions. Emerging technologies, including co-amorphous systems, SCF processing, and 3D printing, provide opportunities for precise control over physicochemical properties and release kinetics. For instance, 3D printing enables the fabrication of complex structures with customizable composition and architecture, especially using ASD materials [374]. Furthermore, the integration of artificial intelligence and machine learning into formulation development could enable predictive modeling of solubility, stability, and bioavailability, thereby reducing empirical trial and error approaches. Over the past decade, ensemble machine learning models such as Random Forest, Extremely Randomized Trees, and Gradient Boosting Regression Trees have been widely used to predict drug solubility in scCO_2_ [375]. Future efforts should also prioritize targeted delivery and personalized medicine strategies, particularly for phytochemicals, where variability in composition and pharmacokinetics remains a major limitation [376].

Despite extensive research, the formulation of BCS class II and IV drugs and poorly soluble phytochemicals remains constrained by several unresolved challenges. A fundamental limitation lies in the lack of a unified strategy capable of simultaneously enhancing solubility and permeability, particularly for BCS class IV compounds. Many current approaches, while effective in improving dissolution, fail to translate into proportional in vivo bioavailability gains due to permeability barriers or rapid precipitation. Additionally, the physical and chemical instability of amorphous systems and nanocarriers, manifested as recrystallization, phase separation, or aggregation, continues to compromise long-term performance. Regulatory challenges persist due to evolving frameworks for nanocarrier-based solubility enhancement strategies, while stringent chemistry, manufacturing, and controls requirements hinder their clinical translation [377]. From a manufacturing perspective, the scalability, reproducibility, and cost-efficiency of advanced systems such as nanosuspensions, SEDDS, and lipid-based carriers remain inadequately addressed. Regulatory uncertainties surrounding novel excipients, complex delivery platforms, and phytochemical formulations further hinder clinical translation, compounded by the absence of standardized evaluation frameworks. Moreover, safety concerns, including potential toxicity of nanomaterials and long-term exposure to excipients, require more rigorous investigation [378].

## 12. Conclusions

In conclusion, despite advances in formulation strategies for poorly water-soluble drugs (PWSDs), including BCS class II/IV compounds and phytochemicals, existing approaches remain fragmented and lack translational consistency. Technologies such as SDs, lipid-based systems, CD complexation, hydrotropy, and nanocarrier-based delivery have demonstrated promise. However, their success is frequently limited by stability, scalability, and regulatory challenges. A paradigm shift toward rational, systems-based formulation design integrating physicochemical, biopharmaceutical, and computational insights is essential to address these challenges. Emerging developments will depend on the ability to bridge the gap between in vitro performance and in vivo outcomes, ensuring that formulation innovations translate into clinically meaningful improvements in bioavailability and therapeutic efficacy.

## Figures and Tables

**Table 1:** Representative carriers used in solid dispersion systems, highlighting their functional roles and associated drug examples.

Excipient	API	Effect	Remark	Reference
Sugars (glucose, galactose)	Sulfamethoxazole	Improved wettability and dissolution	Suitable for eutectic systems; may recrystallize	[73]
Sugars (dextrose, galactose, sucrose)	Corticosteroids	Enhanced dissolution rate	Useful for taste masking	[74]
Sugar alcohols (mannitol, sorbitol, xylitol)	Carbamazepine	Improved solubility and dissolution	Effective in thermal processing methods	[75]
Lactose, galactose	Carbamazepine, ethenzamide	Enhanced dissolution	Acts as a low molecular weight carrier	[76]
Trehalose	Ketoconazole	Increased dissolution rate	Suitable for spray drying techniques	[77]
Organic acids (citric, tartaric, malic, glutaric)	Itraconazole	Improved solubility via pH modification	Enables co-amorphous systems	[78]
Fumaric acid	Naftopidil	Enhanced dissolution and absorption	Drug–acid interaction improves performance	[79]
Succinic acid	Griseofulvin	Improved dissolution	Early example of a solid dispersion system	[80]
Polyvinylpyrrolidone (PVP)	Lansoprazole	Enhanced dissolution and stability	Prevents recrystallization	[81]
Poly(vinylpyrrolidone-vinyl acetate) (PVA/VA)	Dronedarone HCl	Improved dissolution rate	Polymer–drug interaction critical	[82]
Polyethylene glycol (PEG)	Rofecoxib	Enhanced solubility	Common carrier in solid dispersions	[83]
Hydroxypropyl methylcellulose (HPMC)	Nitrendipine	Improved stability and dissolution	Controls crystallization rate	[84]
Cyclodextrins	Telmisartan	Increased solubility via complexation	Dependent on CD type	[85]
Soluplus^®^	Lopinavir	Improved solubility and bioavailability	Polymeric surfactant system	[86]
HPMC	Nifedipine	pH-dependent release	Protects the drug from gastric degradation	[87]
Eudragit^®^ L100-55	Efonidipine HCl	Stabilized supersaturation	Enhances oral absorption	[88]
Poloxamer 188	Paclitaxel	Improved dissolution and bioavailability	Surfactant-based stabilization	[89]
Sodium lauryl sulfate	Tacrolimus	Enhanced bioavailability	Improves wetting and solubilization	[90]
Gelucire^®^	Carbamazepine, Tolbutamide	Improved dissolution	Lipid-based carrier with surfactant properties	[91]
Glyceryl monostearate	Docetaxel	Enhanced solubilization	Suitable for lipophilic drugs	[92]
Stearic acid	Ibuprofen	Improved stability and dissolution	Lipid matrix system	[93]
Nicotinamide	Quetiapine fumarate	Enhanced dissolution via a co-amorphous system	Disrupts the crystal lattice	[94]

**Table 2:** Summary of studies on amorphous solid dispersion-based formulations, highlighting formulation approaches, drugs, and key outcomes.

Technique/Excipient	API	Effect	Remark	Reference
KinetiSol^®^/Hydroxypropyl methylcellulose acetate succinate (HPMCAS)	Abiraterone acetate	12.4–13.8-fold increase in bioavailability	Effective for poorly soluble Class IV drugs; ternary systems show added benefit	[152]
Kneading/Soluplus^®^	Apixaban	5.9-fold solubility and 2.54-fold permeability increase; 2.31-fold bioavailability improvement	Enhances both solubility and permeability	[153]
Solvent evaporation/PVP, PVP/VA, polyacrylic acid (PAA), HPMCAS	Carbamazepine	Improved physical stability via intermolecular interactions	Requires a balance between physical and chemical stability	[154]
Solvent evaporation/PVP/VA, HPMC	Curcumin	Significant solubility increase (up to ~315 µg/mL)	No major impact on permeability	[155]
Solvent impregnation/evaporation/poly(2-hydroxyethyl methacrylate) (PHEMA), PVA, PVP	Fenofibrate	Enhanced absorption with polymer-dependent release profiles	PVP systems show faster release than PVA/PHEMA	[156]
Spray anti-solvent precipitation/HPMCP, HPMCAS	Lumefantrine	Markedly improved drug release (140 µg/mL vs. <80 ng/mL)	Superior to marketed formulation (Coartem^®^)	[157]

**Table 3:** Overview of common cosolvents in pharmaceutical formulations, highlighting their polarity, applications, advantages, and limitations.

Cosolvent	Polarity	Typical Use	Advantages	Limitations	Reference
Acetone	Polar aprotic	Experimental studies	Effective cosolvency behavior	Not suitable for final dosage forms	[212]
Benzyl alcohol	Moderate polarity	Parenterals (also preservatives)	Dual role as cosolvent and preservative	Neonatal toxicity risk	[212]
Benzyl benzoate	Low–intermediate	Injectable oily solutions	Enhances solubility in depot systems	Irritation; restricted concentration	[212]
Diethylene glycol monoethyl ether (Transcutol^®^)	Intermediate	Topical, transdermal, nanoformulations	Excellent solubilizer and permeation enhancer	Limited parenteral use	[213]
Dimethyl sulfoxide	Highly polar aprotic	Preclinical research formulations	Strong solubilizing ability	Toxicity limits clinical use	[214]
Dimethylacetamide	Polar aprotic	Depot injections (limited)	Good for poorly soluble drugs	Regulatory and toxicity limitations	[215]
Ethanol	Intermediate	Elixirs, tinctures, injectables	Widely accepted, good solubilizer	Pediatric restriction, inflammability, volatility	[216]
Glycerin	Highly polar	Oral liquids	Safe, sweet taste	Limited solubilization power	[217]
Isopropyl alcohol	Intermediate	Topical formulations	Good solvent	Not used in internal dosage forms	[218]
N-Methyl-2-pyrrolidone	Polar aprotic	Parenteral and depot systems (limited)	Strong solubilization capacity	Toxicity concerns, restricted levels	[219]
Polyethylene glycol 200/300	Polar	Oral, topical	Lower viscosity than PEG 400	Osmotic effects at high dose	[220]
Polyethylene glycol 400	Polar	Oral liquids, soft gels	Good solubilizing capacity	Viscosity issues	[221]
Propylene glycol	Polar	Oral and parenteral	Solubilizer, low toxicity, stable	High concentrations may cause irritation	[222]
Triacetin (Glyceryl triacetate)	Intermediate	Oral and injectable (limited)	Good solubilizer for lipophilic drugs	Limited water miscibility at high levels	[223]

**Table 4:** Summary of hydrotrope-based formulation approaches, including techniques/excipients, drugs, effects, and remarks.

Technique/Excipient (Hydrotrope)	API	Effect	Remark	Reference
Arginine	Bezafibrate	Enhanced solubility and 255% increase in oral bioavailability	Hydrogen bonding and amorphization improve drug release	[235]
Urea, nicotinamide	Carbamazepine	~30-fold solubility increase	Significant permeability reduction (solubility–permeability trade-off)	[236]
Amino acids (e.g., phenylalanine, tryptophan, arginine, proline, valine, glycine)	Carbamazepine, indomethacin	Up to 7-fold solubility increase	Additive effects via hydrogen bonding and π–π interactions	[237]
Sodium citrate + N,N-dimethylurea	Diclofenac sodium	High solubility (~63 mg/mL)	Synergistic effect; solvent-free and sustainable approach	[238]
Sodium benzoate + nicotinamide	Glibenclamide	Increased solubility (10–40% systems)	Blends are more effective; apparent permeability reduction is observed	[239]
Nicotinamide, caffeine, urea	Lidocaine, procaine, benzocaine	Improved aqueous solubility	Enhancing molecular interactions; nicotinamide is most effective; supported by thermodynamic modeling	[240]
Sodium salicylate	Mefenamic acid	Up to 22.9-fold solubility increase	Strong concentration-dependent solubilization	[241]
Urea, mannitol, citric acid, sodium benzoate, sodium salicylate	Rosuvastatin calcium	Near-complete drug release (~99–105%)	Combination systems more effective than single hydrotropes	[242]

**Table 5:** Compilation of patented hydrotropic systems, outlining formulation approaches, associated APIs or systems, observed effects, key remarks, and patent identifiers.

Technique/Excipient (Hydrotrope)	API/System	Effect	Remark	Patent No.
Organic acids + β-CD (inclusion–hydrotropy–recrystallization)	Chlortetracycline hydrochloride	Enhanced solubility and inclusion complex formation	Suitable for heat-sensitive and aqueous-unstable drugs	112957481
Hydrotropic solid dispersion	Lipophilic drugs (topical gels)	Improved aqueous solubility and bioavailability	Enhances formulation efficiency for topical delivery	2021106377
Cyclodextrin as a hydrotrope	Lipid compounds (fatty acids, vitamins, cholesterol)	Complete aqueous solubilization	Eliminates the need for organic solvents; suitable for biological systems	116200325
Amino compounds + Resorcinol	Dibromohydantoin	Rapid and complete dissolution	Synergistic hydrotropy via hydrogen bonding and chemical interaction	116868999
Hydrotrope + polyol/oil solvent system	Poorly soluble drugs	Improved solubilization	Versatile composition for multiple APIs	102643474
Polyols + hydrotropic additives	Ferulic acid	Enhanced solubility and stability	Suitable for skincare/cosmetic applications	118304206
Hydrotropy-assisted nanoemulsion	Vitamin D and analogs	Improved solubility, stability, and bioavailability	Nanoencapsulation (<500 nm) in dry powder form	118750454
Hydrotropic monomers in a polymer matrix	Sodium sarcosinate	Controlled/slow drug release	Reduces burst release and dosing frequency	119587708

**Table 6:** Overview of recent cyclodextrin-based inclusion systems, including techniques/excipients, drugs, effects, and remarks.

Technique/Excipient (CD System)	API	Effect	Remark	Reference
Methyl-β-cyclodextrin (CD)	Albendazole	~150,000-fold solubility increase; ~90% release in 10 min	Improved pharmacokinetics (↑ Cmax, AUC)	[268]
Heptakis(2,6-di-O-methyl)-β-CD, hydroxypropyl-β-CD (HP-β-CD), Sulfobutylether-β-CD (SBE-β-CD), randomly methylated-β-CD (RM-β-CD)	Apixaban	Up to ~78.7-fold solubility increase (1968.7 μg/mL)	Strong inclusion confirmed by molecular docking	[269]
Methyl-β-CD	Astilbin	Solubility up to 43 mg/mL; improved dissolution	~11.9-fold increase in oral bioavailability	[270]
Heptakis(2,6-di-O-methyl)-β-CD	Carvedilol	~26.47-fold solubility increase	Stable 1:1 inclusion complex	[271]
HP-β-CD, β-CD	Ethanamizuril	~21.9-fold solubility increase	HP-β-CD shows higher stability and efficiency	[272]
β-CD	Itraconazole	~4-fold solubility enhancement	Improved corneal permeation; non-irritant	[273]
HP-β-CD (optimal)	Ivacaftor	~40–100-fold solubility increase	Improved bioavailability and sustained release	[274]
Methyl-β-CD	Ketoprofen	Significant solubility enhancement	1:1 inclusion complex formation	[275]
HP-β-CD, methyl-β-CD, SBE-β-CD	Naproxen	Enhanced antimicrobial activity	High stability constants (~1123–1261 M^−1^)	[276]
SBE-β-CD	Nimodipine	~1202-fold solubility increase	Improved therapeutic outcomes vs. marketed product (Nimotop^®^ injection)	[277]
β-CD	Paclitaxel	Enhanced solubility and >95% entrapment	Improved cytotoxicity (anticancer activity)	[278]
β-CD, HP-β-CD, methyl-β-CD	Ribavirin	Rapid drug release (~93% in 10 min)	Methyl-β-CD showed the highest stability	[279]
HP-β-CD	Rosuvastatin calcium	2–6-fold solubility increase	~3-fold bioavailability increase with IVIVC	[280]
β-CD	Voriconazole	Solubility increased to 70.64 mg/mL	Improved dissolution (~98.47%)	[281]

**Table 7:** Recent cyclodextrin-based inclusion systems for phytochemicals, outlining formulation approaches, effects, and relevant remarks.

Technique/Excipient (CD System)	Phytochemical	Effect	Remark	Reference
HP-β-CD ± chitosan (ternary complex)	Apigenin	>8-fold solubility increase; improved dissolution and bioactivity	Enhanced antioxidant and anti-inflammatory activity	[282]
HP-β-CD	Baicalein	Increased solubility (147.38 μg/mL)	Improved antioxidant and antibacterial activity	[283]
HP-β-CD (binary/ternary)	Chrysin	Up to ~99% drug release; higher stability constant	A ternary system is more stable than a binary system	[284]
β-CD	Curcumin	~205-fold solubility enhancement	Improved anticancer activity (apoptosis, reduced migration)	[285]
HP-β-CD	Hesperidin, Hesperetin	>1000–2000-fold solubility increase	Enhanced antioxidant and enzyme inhibition activity	[286]
HP-β-CD	Kaempferol	~5.45-fold solubility increase	Reduced lipophilicity and improved permeability	[287]
β-CD ± Pluronic F127	Luteolin	6–10-fold solubility increase	Improved antioxidant activity	[288]
HP-β-CD	Quercetin, Rutin	~630-fold (quercetin) and ~55-fold (rutin) increase	Enhanced dissolution and antiproliferative activity	[289]
SBE-β-CD	Resveratrol	~66-fold solubility increase	Improved stability, cellular uptake, and cytotoxicity	[290]
β-CD	Tetrahydrocurcumin	~65-fold solubility enhancement	Improved tumor regression in vivo	[291]

**Table 8:** Nanocarrier-based techniques used for solubility enhancement of active pharmaceutical ingredients (APIs), along with their effects and remarks.

Technique/Excipient (Nanocarrier)	API	Effect	Remark	Reference
Nanocrystals/Nanosuspensions	Fenofibrate	Enhanced dissolution and saturation solubility	Particle size reduction increases surface area; suitable for multiple routes	[330]
Polymeric nanoparticles	Docetaxel	Improved solubility and stability	Enables controlled release and targeted delivery	[331]
Nanosponges	Domperidone	Enhanced solubility and stability	High drug loading and controlled release	[332]
Liposomes	Paclitaxel	Improved solubility and reduced toxicity	Biocompatible with targeting capability	[333]
Polymeric micelles	Combretastatin derivatives	Enhanced solubility and permeability	Amphiphilic self-assembly improves drug loading	[334]
Solid lipid nanoparticles (SLNs)	Cilostazol	Improved dissolution and sustained release	Biocompatible with enhanced bioavailability	[335]
Nanostructured lipid carriers (NLCs)	Ranolazine	Increased solubility and drug loading	Higher stability and bioavailability than SLNs	[336]
Nanoemulsions	Phenytoin	Enhanced solubility and absorption	Large interfacial area improves membrane transport; versatile routes	[337]
Lipid–polymer hybrid nanoparticles	Tetrahydrocurcumin	Improved solubility and controlled release	Combines the advantages of lipid and polymer systems	[338]

**Table 9:** Summary of recent studies on nanocarrier systems combined with cyclodextrins for enhancing the solubility of poorly aqueous-soluble APIs.

Technique/Excipient (Nanocarrier + CD)	API	Effect	Remark	Reference
Liposomes + HP-β-CD	Pinostilbene	~10-fold solubility increase; reduced degradation (<15%)	Improved encapsulation and controlled release	[355]
Nanosponges (β-CD)	Curcumin	~2.95-fold solubility increase	Higher stability constant than conventional β-CD complexes	[356]
Solid lipid nanoparticles (SLNs) + HP-β-CD	Nebivolol	Improved solubility and oral delivery	Pre-complexation enhances encapsulation and dispersibility	[357]
Nanostructured lipid carriers (NLCs) + SBE-β-CD	Besifloxacin HCl	~8-fold solubility increase; sustained release (~8 h)	Improved corneal permeation vs. the marketed product	[358]
Self-nanoemulsifying drug delivery systems + HP-β-CD	Insulin	Enhanced solubilization and stability	Reduced burst release; improved oral bioactivity	[349]
Functionalized nanoparticles + β-CD	Quercetin	Controlled/light-responsive release	Reduced toxicity and improved delivery efficiency	[359]
CD-metal–organic frameworks (γ-CD)	Quercetin	~100-fold solubility increase	Improved antioxidant activity and reduced cytotoxicity	[360]
Nanogels (SBE-β-CD)	Flurbiprofen	Controlled release; reduced permeability	Sustained drug delivery with lower flux	[361]
Polymeric micelles (CD derivatives)	Cannabidiol, Tetrahydrocurcumin	Enhanced solubility and biocompatibility	Low cytotoxicity in endothelial cells	[362]
Electrospun nanofibers (HP-β-CD)	Acyclovir	Faster dissolution; ~98% drug loading	Improved disintegration compared to PVP systems	[363]

## Data Availability

No new data were created or analyzed in this study.

## Abbreviations

The following abbreviations are used in this manuscript:

APIs	Active pharmaceutical ingredients
ASDs	Amorphous solid dispersions
BCS	Biopharmaceutics classification system
CD	Cyclodextrin
DESs	Deep eutectic solvents
GIT	Gastrointestinal tract
HME	Hot-melt extrusion
HP-β-CD	Hydroxypropyl-beta-cyclodextrin
HPMC	Hydroxypropyl methylcellulose
HPMCAS	Hydroxypropyl methylcellulose acetate succinate
HPMCP	Hydroxypropyl methylcellulose phthalate
IVIVC	In vitro–in vivo correlation
Mβ-CD	Methyl-β-cyclodextrin
NLCs	Nanostructured lipid carriers
PEG	Polyethylene glycol
PVA	Polyvinyl alcohol
PVA/VA	Poly(vinylpyrrolidone-vinyl acetate)
PVP	Polyvinylpyrrolidone
PWSD	Poorly water-soluble drug
RCM	Reaction cocrystallization method
SD	Solid dispersion
SEDDS	Self-emulsifying drug delivery systems
SFD	Spray freeze drying
SFCL	Spray-freezing into cryogenic liquid
SLNs	Solid lipid nanoparticles
SBE-β-CD	Sulfobutylether-beta-cyclodextrin
scCO_2_	Supercritical carbon dioxide
SCF	Supercritical fluid
Tg	Glass transition temperature
THEDES	Therapeutic deep eutectic systems
TFF	Thin film freezing
XRD	X-ray diffraction
